# Genome Wide MeDIP-Seq Profiling of Wild and Cultivated Olives Trees Suggests DNA Methylation Fingerprint on the Sensory Quality of Olive Oil

**DOI:** 10.3390/plants10071405

**Published:** 2021-07-09

**Authors:** Oussama Badad, Naoufal Lakhssassi, Nabil Zaid, Abdelhalim El Baze, Younes Zaid, Jonas Meksem, David A Lightfoot, Huseyin Tombuloglu, El Houcine Zaid, Turgay Unver, Khalid Meksem

**Affiliations:** 1 Department of Plant, Soil and Agricultural Systems, Southern Illinois University, Carbondale, IL 62901, USA; oussama.badad@gmail.com (O.B.); naoufal.lakhssassi@siu.edu (N.L.); abdelhalim.elbaze@siu.edu (A.E.B.); ga4082@siu.edu (D.A.L.); 2 Department of Biology, Faculty of Sciences, Mohammed V University, Rabat 10000, Morocco; zaidnabiiil@gmail.com (N.Z.); younes_zaid@yahoo.ca (Y.Z.); ijazaid@gmail.com (E.H.Z.); 3 Research Center, Abulcasis University of Health Sciences, Rabat 10000, Morocco; 4 Trinity College of Arts and Sciences, Duke University, Durham, NC 27708, USA; jmeksem@gmail.com; 5 Department of Genetics Research, Institute for Research and Medical Consultations (IRMC), Imam Abdulrahman Bin Faisal University, P.O. Box 1982, Dammam 31441, Saudi Arabia; htoglu@iau.edu.sa; 6 Ficus Biotechnology, Ostim OSB Mah, 100. Yil Blv, No:55, Yenimahalle, Ankara 06000, Turkey

**Keywords:** olive epigenomic, differential methylation, olive genetic diversity, *Olea europaea ssp. europaea var. sylvestris*, aromatic volatile compounds

## Abstract

Secondary metabolites are particularly important to humans due to their pharmaceutical properties. Moreover, secondary metabolites are key compounds in climate change adaptation in long-living trees. Recently, it has been described that the domestication of *Olea* subspecies had no major selection signature on coding variants and was mainly related to changes in gene expression. In addition, the phenotypic plasticity in *Olea* subspecies was linked to the activation of transposable elements in the genes neighboring. Here, we investigated the imprint of DNA methylation in the unassigned fraction of the phenotypic plasticity of the *Olea* subspecies, using methylated DNA immuno-precipitation sequencing (MeDIP-seq) for a high-resolution genome-wide DNA methylation profiling of leaves and fruits during fruit development in wild and cultivated olives from Turkey. Notably, the methylation profiling showed a differential DNA methylation in secondary metabolism responsible for the sensory quality of olive oil. Here, we highlight for the first time the imprint of DNA methylation in modulating the activity of the Linoleate 9S lipoxygenase in the biosynthesis of volatile aromatic compounds. Unprecedently, the current study reveals the methylation status of the olive genome during fruit ripening.

## 1. Introduction

Olive (*Olea europaea*) oil is a major component of the Mediterranean diet and is associated with multiple health benefits. Although extra virgin olive oil (EVOO) is primarily made of triacylglycerols, an abundance of phenolic and volatile compounds are also present [1]. Phenolic compounds are secondary plant metabolites, a major group of plant compounds chemically characterized by the presence of one or more aromatic rings [2]. The olive tree (*O. europaea*) produces a large arsenal of polyphenols that includes phenylpropanoids, terpenoids, and the most important and abundant subclass of phenolic compounds in olive oil: the secoiridoids such as oleuropein and its derivatives. The polyphenols in olive oil have multiple pharmaceutical properties such as fighting inflammation, coronary heart diseases, and several types of cancers [3]. In Turkish groves, particularly in the Aegean coast, *Ayvalik cv* is the most grown cultivar and is used mainly for oil production for its sensory qualities. *Ayvalik cv* olive oil has been extensively characterized and is appreciated for its mild fruity flavor due to low phenolic content varying from 106 to 296 mg/kg [4,5]. In contrast, oil extracted from wild olives is less encountered because of the bitterness and peppery taste caused by its high volatile aromatic and phenolic content. The analysis of wild olive oil from Tunisia, Algeria, Portugal, and Spain showed that their total phenolic content varies from 340 to 870 mg/kg [6,7,8,9]. In a genetic diversity study on wild olives and *Ayvalik cv* from the Aegean region of Izmir, *Ayvalik cv* showed a similarity of 97% with the wild olives [10]. The results from this study raised the question about the process by which genetically close subspecies can have a significant difference in their phenotypes, mainly, their observed phenolic composition in this case.

After the release of the first assembled and annotated chromosome level wild olive (*Olea europaea ssp. europaea var. sylvestris*) genome sequence [11], which uncovered the genetic code behind the health benefits of olive oil. The importance of the olive tree in the Mediterranean diet and economy raised the interest to explore beyond the boundaries of the DNA code. Interestingly, due to the siRNA extraordinary regulation of the fatty acid desaturase-2 (*FAD2*) gene family expression in favor of the accumulation of the oleic acid in the olive oil [11], several questions were raised about the possible existence of another type of epigenetic regulation: DNA methylation for instance. Notably, epigenetic regulation is important for environmental adaptation by modulating gene expression as it has been suggested to accelerate adaptive responses to selection process [12,13,14].

The genetic diversity of olive has been extensively investigated. In earlier studies, olive cultivars were mainly clustered based on agronomical traits such as size, the shape of fruits, pits, leaves, general stature of the tree, oil, and polyphenolic content. With the current advance in molecular biology and sequencing technologies, the scientific community dug deeper to understand other aspects of the genetic diversity using molecular markers, beginning with random amplified polymorphic DNA (RAPD) [15], chloroplastic DNA markers [16], mitochondrial DNA and RAPD [17], allozyme variation [18], amplified fragment length polymorphism (AFLP) [19], and simple-sequence repeats (SSR) [20]. Recently, Besnard et al. have revealed the presence of three main lineages of pre-Quaternary origin by performing the phylogeographic and Bayesian molecular dating analyses based on plastid genome profiling of wild and cultivated genotypes [21]. Moreover, an evolutionary transcriptomic study on wild and cultivated olive trees showed no major signatures of selection on coding variants in addition to weak signals that are primarily affected by transcription factors. Interestingly the phylogenetic analysis based on gene expression could not discriminate the wild and cultivated accessions from the eastern part of the Mediterranean including the Turkish accessions [22]. Furthermore, the transposable elements activity in the olive genome was shown to be involved in generating genetic variability in cultivated olives [23].

Epigenetics is a word coined by Waddington in 1942, defined by the inherited somatic changes that are not associated with changes in DNA sequences [24]. DNA methylation is one of the most studied epigenetic marks that can be inherited during cell division [25], and can mediate epigenetic regulation of gene expression. This regulation is due to the recruitment of proteins involved in gene repression or inhibition of the binding of transcription factors to DNA [26]. Methylation of cytosine residues in DNA is enzymatically catalyzed by DNA methyltransferases, which transfer a methyl group from *S*-adenosyl-L-methionine to the 5-position [27]. DNA methylation occurs in different genomic features including CpG islands, intergenic regions, gene bodies, and gene promoters for different functionalities. For instance, methylation of transcription binding sites leading to a suppression of gene expression can lead to a higher level of gene expression when the methylation is located in the gene body [28]. The evolution of NGS based technologies such as endonuclease digestion and bisulfite conversion made genome-wide DNA methylation mapping pretty feasible, but it remains relatively expensive. Affinity enrichment analysis such as MeDIP-seq Generates unbiased, cost-effective, and full-genome methylation levels without the limitations of restriction sites or CpG islands. To date, DNA methylation has been studied in many plant species, including algae [29], cereal crops [30], vegetables [31,32,33]; however, no studies were conducted on olive trees.

Prominent evidence suggests that DNA methylation plays an important role in the ripening of climacteric fruits. In tomatoes (*Solanum lycopersicum*), the genome undergoes a global decrease in methylation levels during ripening due to the increased expression of DNA demethylases [34]. The downregulation of the *RdDM* gene during fruit ripening in non-climacteric strawberries (*Fragaria ananassa*) resulted in ripe fruits with a lower DNA methylation level than immature fruits, suggesting a decrease in DNA methylation during ripening of non-climacteric fruits [35]. Recently, a study on DNA methylation in orange (*Citrus sinensis*) a non-climacteric fruit showed a global increase of DNA methylation with fruit ripening. These previous studies raised the question about whether or not the DNA methylation behavior during fruit ripening is related to the fruit ripening mechanism.

In addition to DNA methylation, phenolic and aromatic volatile compounds have been involved in ripening. In apple (*Malus x domestica*), 6 out of 22 putative LOX genes are expressed in a ripening dependent manner. The previous study showed that the activity of 9-LOX gene was related to linoleic and linolenic acids hydroperoxidation: a crucial step in the biogenesis of six-carbon volatile aldehydes, which are major constituents of the aroma in extra virgin olive oil (EVOO) [36]. In pepper (*Capsicum annuum*), *9-LOX* (*CaLOX1*) gene, encodes a 9-specific lipoxygenase that catalyzes the hydroperoxidation of linoleic acid [37]. In olive, two lipoxygenases are characterized and at least three copies of *Oep1LOX2* and one copy of *Oep2LOX2* are present in the olive genome [38]. Besides volatile compounds, polyphenols play a major role in fruit defense during early ripening and also provide characteristic color (anthocyanins) to ripened fruits [39]. Phenolic content in olive fruits is high during early maturation stage and decreases to reach the lowest when fruits are ripe and ready to be harvested. The phenolic content is modulated by the activity of Peroxidase (*POX*), β-glucosidase (*GLU*) gene families [40]. Interestingly the beta glucosidase gene family gene expression has been described as high in early maturation stage and decrease with fruit ripening [41].

The current study aims to elucidate and bring more insight into DNA methylation dynamic and its fingerprint on the important properties of olives and their by-product by comparing the landscape between wild and cultivated olive trees.

## 2. Results

### 2.1. Evolution of DNA Methylation Landscape across Olive Tree Tissues

To investigate the evolution of DNA methylation landscape during fruit ripening across tissues (leaves and fruits) in the wild and cultivated olive trees, the differential methylation in different genomic features including 1 KB promoter, untranslated regions (UTRs), introns, and exons were analyzed. The results show that the global levels of methylation in the leaves were higher for all studied genomic features when compared to the fruit in both cultivated and wild olives during fruit ripening (Figure S1A–D).

To further explore the relationship between DNA methylation behavior and ripening in both cultivated and wild olives. The difference in methylation status in leaves and fruits between different maturity stages was called using MACS2. The results showed a global decrease in methylation level in the fruit with ripening for both wild and cultivated olives including all 23 olive chromosomes. Interestingly the hypomethylation was uniform in the wild *Olea europaea ssp. europaea var. sylvestris* chromosomes where all the 23 pairs manifested a decrease in methylation level unlike the cultivated *Ayvalik cv* (Figure S2). Intriguingly, the hypomethylation with fruit ripening was significant in the wild *Olea europaea ssp. europaea var. sylvestris* when compared to the cultivated *Ayvalik cv* (Figure S3A,B). However, DNA methylation manifested a different trend in the leaves, where a global increase in methylation level was observed in the wild and cultivated leaf samples (Figure S3C,D).

### 2.2. Domestication Fingerprint on DNA Methylation Landscape

In order to bring insight into the domestication fingerprint on DNA methylation in *Olea* subspecies in the Aegean region, the methylomes of the cultivated and wild olives were compared for both leaves and fruits in July (early maturity phase) and November (advanced maturity phase). When comparing the methylation profiles of the fruits in early maturation stage, the global methylation level was shown to be relatively higher in the cultivated *Ayvalik cv* for all the genomic features except around the transcription termination site (Figure S4A). During advanced fruit maturation stage, the methylation manifested an opposite behavior and became higher in the wild olives in all genomic features (Figure S4B). In the early maturation phase, no major significant difference in global levels of methylation were observed in the leaves. However, differences were observed in the distribution of methylated regions across the genomic features. Interestingly, in July, the level of methylation was higher in the transcription regulatory regions (promoter, TSS, and TTS) of the wild *Olea europaea ssp. europaea var. sylvestris*, while the methylation of gene body (intron and exon) was higher in the cultivated *Ayvalik cv* (Figure S4C). In November, the methylation level in the leaves of the wild olives was significantly higher in all genomic features (including TSS, TTS, intron, and exon) when compared to the cultivated *Ayvalik cv* (Figure S4D). 

### 2.3. Functional Enrichment Analysis for Genes Associated with Differentially Methylated Regions

To examine the potential functionality of genes associated with methylated regions in the cultivated *Ayvalik cv* and *Olea europaea ssp. europaea var. Sylvestris* genomes, enrichment analysis for gene ontology terms (molecular function, cellular component, and biological process) and KEGG enriched pathways were performed using HOMER (Table S4).

**Figure 1:**
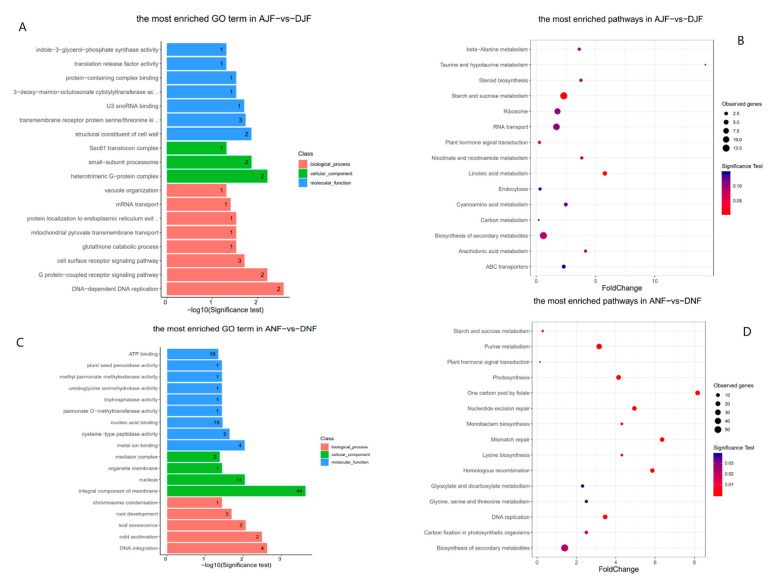
Enrichment analysis of methylated regions in cultivated and wild olives. (**A**) Most enriched gene ontology terms in methylated regions in both cultivated and wild fruits in July. (**B**) Most enriched KEGG pathways in methylated region in both cultivated and wild fruits in July. (**C**) Most enriched gene ontology terms in methylated region in both cultivated and wild November fruits. (**D**) Most enriched KEGG pathways of methylated regions in both cultivated and wild November fruits. ANF: Ayvalik November fruit, DNF: wild November fruit. AJF: Ayvalik July fruit, DJF: wild July fruit, where Delice is the Turkish name of the wild oleaster.

In the fruit samples, the results showed that genes associated with methylated regions were mostly enriched in gene ontology terms related to the following molecular function and biological processes: ripening of non-climacteric fruit (methyl jasmonate methylesterase activity, jasmonate O–methyltransferase activity), binding (nucleic acid binding, ATP binding, U3 snoRNA binding), and transport activity (mRNA transport, mitochondrial pyruvate transmembrane transport). To gain more insights into DNA methylation’s role in modulating important metabolic activity, KEGG enrichment analysis revealed the potential involvement of genes associated with methylated regions in six major categories of metabolism pathways, including amino acid metabolism, secondary metabolite metabolism (steroid, and phenylpropanoid), carbohydrate metabolism, energy metabolism (photosynthesis), lipid metabolism (alpha–Linolenic acid metabolism, Linoleic acid metabolism, arachidonic acid metabolism, glycerolipids, and sphingolipid), and metabolism of co-factors and vitamins (Figure 1). Due to the high number of entities, only the fifteen most enriched pathways are shown in the GO bar charts and KEGG enrichment scatter plots.

**Figure 2:**
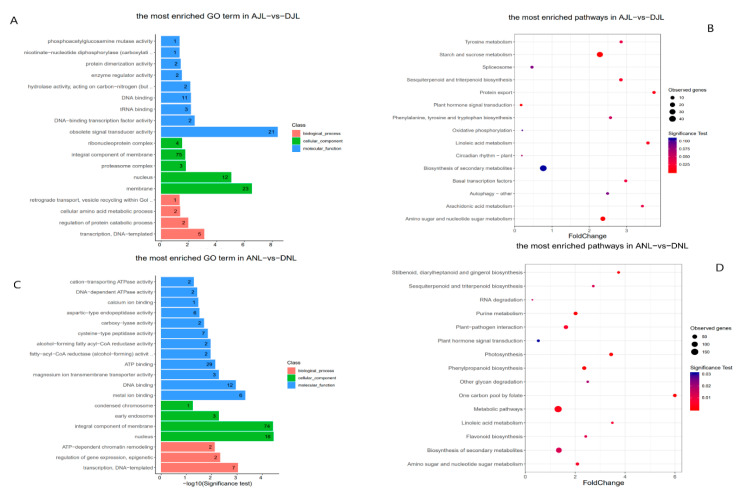
Enrichment analysis of methylated regions in cultivated and wild olives. (**A**) Most enriched gene ontology terms in methylated regions in both cultivated and wild olives leaf samples in July. (**B**) Most enriched KEGG pathways in methylated region in both cultivated and wild olive leaf samples in July. (**C**) Most enriched gene ontology terms in methylated region in both cultivated and wild olives leaf samples in November. (**D**) Most enriched KEGG pathways of methylated regions in both cultivated and wild olives leaf samples in November. ANL: Ayvalik November fruit, DNL: wild November fruit. AJL: Ayvalik July fruit, DJL: wild July fruit, where Delice is the Turkish name of the wild Oleaster.

In the leaf samples, the majority of genes associated with methylated regions were enriched in gene ontology terms related to transcriptional activity, amino acid metabolism, and transport activity (Figure 2A,C). The KEGG enrichment analysis (Figure 2B,D) showed the potential involvement of the majority of genes associated with methylated regions in secondary metabolic pathways especially in some of the most important secondary metabolisms in olives including phenylpropanoid, flavonoid, Stilbenoid, and sesquiterpenoid and triterpenoid biosynthesis. We observed an increase of differentially methylated genes number with the fruit maturity as well as the significance of the test.

### 2.4. Comparative Methylated Promoter Analysis between Wild and Cultivated Olives

Putative genes involved in fatty acid and volatile aromatic compounds metabolism were retrieved from the literature [11,36] and mapped against the wild olive genome annotation database to extract methylated gene IDs.

**Figure 3:**
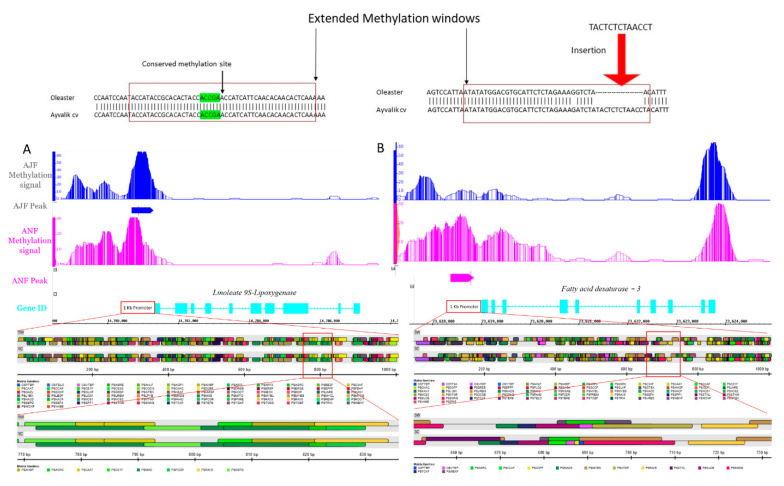
Comparative promoter analysis of methylated putative genes responsible for the sensory and organoleptic qualities of olive oil between wild and cultivated olives. (**A**): linoleate 9S-Lipoxygenase. (**B**): Fatty acid desaturase FAD3. Colored boxes indicate the cis-elements in each promoter region. Zoomed parts show conserved, gained, and/or lost cis-elements at the methylation window between wild and cultivated olives. AJF: Ayvalik July fruit, ANF: Ayvalik November fruit, DNF: Delice November fruit sample and DJF: Delice July fruit sample.

The alignment of the wild *Olea europaea ssp. europaea var. sylvestris* methylated genes (1K promoter) against the cultivated *Ayvalik cv* assembly provided by the international olive genome consortium resulted in alignment identities ranging from 95% for *a putative fatty acid desaturase-3* (Genbank ID MZ361724) to 99% for putative linoleate 9S-lipoxygenase (Genbank ID MZ361723). Pairwise alignment available in (Additional file S1). The results showed a conserved sequence of the methylation window including 24 bp flanking the summit of the peak for linoleate 9S-lipoxygenase and therefore, the conservation of the 1 kb promoter *cis-elements* between wild and cultivated Turkish olives (Figure 3A) (*cis-elements* available in the (Table S5).

In the other hand, the putative *FAD3* promoter sequence pairwise alignment showed the presence of multiple SNPs in addition to an insertion of a 12 nucleotides “TACTCTCTAACC” sequence in the *Ayvalik cv FAD3* promoter (Figure 3B) causing gain of new and loss of ancestral *cis-elements* (Table S6).

### 2.5. Metabolic Profiling of Ayvalik Cv and Wild Olea europaea ssp. europaea var. sylvestris

The gas chromatography analysis revealed a slight difference in the most important fatty acids including oleic acid (C18:1), linoleic acid (C18:2), and linolenic acid (C18:3) content. On the other hand, the HPLC-UV and the NMR analysis showed a significant difference in the total phenolic content between *Ayvalik cv* and the wild olive oil due to the high amount of oleuropein derivatives in the wild EVOO (Table 1).

## 3. Discussion

**Figure 4:**
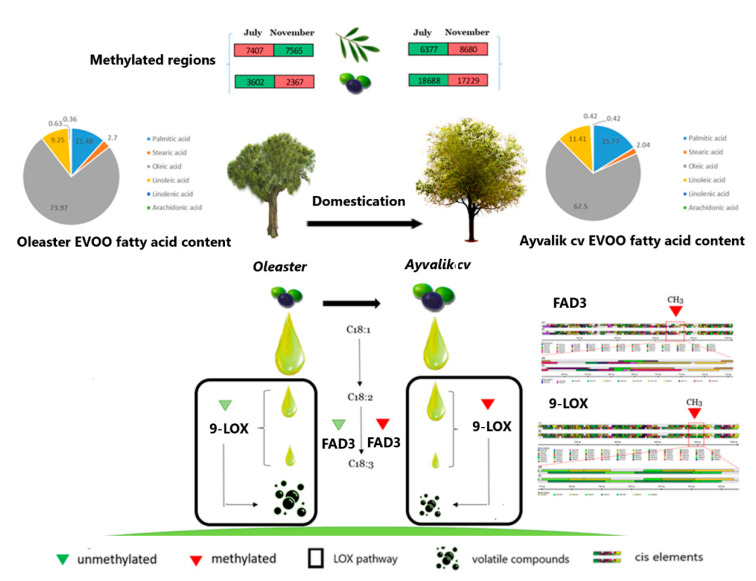
Scheme summarizing the effect of DNA methylation during olive domestication on the sensory quality of olive oil.

DNA methylation and secondary metabolites are key components in constantly changing environment adaption for long-living trees and play an eminent role in fleshy fruit ripening. It has been reported that the genome of non-climacteric fruits undergoes a global decrease of methylation levels during fruit ripening, such as in strawberries [35], while an opposite global increase of DNA methylation was observed in ripened orange fruits [42]. To bring more insight into the evolutionary landscape of DNA methylation in non-climacteric fruits ripening, we studied and compared the genome wide methylation profiling of wild and cultivated olives from turkey in two fruit maturation stages (Figure 4).

### 3.1. Differential Methylation across Tissues

To date, most available studies on methylation focus on comparing different vegetative tissues like leaves, shoots, roots, cotyledons, and inflorescence, but little is known about fruit’s comparative methylation. Previous studies have only reported high levels of methylation in vegetative organs in a small set of plants including the plant model *Arabidopsis thaliana,* tomatoes, and *Silene latifolia* [43,44,45]. The results showed a higher methylation level in the leaves compared to the fruit in both wild *Olea europaea ssp. europaea var. sylvestris* and cultivated olive (Figure S2).

Since the wild *Olea europaea ssp. europaea var. sylvestris* and *Ayvalik cv* manifested the same methylation behavior with ripening, DNA methyltransferase and demethylases gene expressions between July and November were extracted from the wild *Olea europaea ssp. europaea var. sylvestris* differential gene expression data in fruit and leaves between July and November [11]. The results showed the induction of 269 *methyltransferases* in the leaves (Figure S5, Additional file S2), compared to only 164 upregulated *methyltransferases* in the fruits (Figure S6, Additional file S3). This is consistent with previous studies showing that the global methylation levels are higher in vegetative tissues [46].

### 3.2. Global Level of Methylation during Fruit Ripening

DNA methylation plays an important role in the ripening of fleshy fruit such as *S. lycopersicum* (tomatoes), *F. ananassa* (strawberries), and *C. sinensis* (oranges) [32,35,42]. The methylation landscape of non-climacteric fruit genomes however is still debatable. Data shown in (Figure S4) revealed a global decrease of DNA methylation during fruit ripening for both the cultivated and wild ancestor *Olea europaea ssp. europaea var. sylvestris*. The observed decrease of methylation levels is most likely due to the upregulation of 24 demethylase genes in the *Olea europaea ssp. europaea var. sylvestris* fruit (Figure S7 and Additional file S4). These results are in accordance with previous studies in *S. lycopersicum* [47] and *F. ananassa* [35]. Meanwhile, the *C. sinensis* genome manifested a global increase in DNA methylation during fruit ripening [42]. Unlike the fruit, the level of methylation in the leaves during ripening manifested a considerable global increase. This hypermethylation associated with ripening is most likely due to the upregulation of a large number of methyltransferases (up to 269) (Figure S5).

### 3.3. Differential DNA Methylation Potential Role in the Sensory and Organoleptic Properties of Olive Oil

#### 3.3.1. Genes Involved in Polyunsaturated Fatty Acids Metabolism and Their Derivatives Volatile Compounds

The current study highlights the potential role of DNA methylation as an important component in phenotypic plasticity in olives trees. Oleic, linoleic, and linolenic acids are the major mono- and polyunsaturated fatty acids in extra virgin olive oil (EVOO). EVOO with higher levels of omega-9, omega-3, and lower levels of omega-6 are highly valued for their aroma and their health benefits to humans in fighting inflammation, cancer, and coronary heart conditions. To date, the characterization of *FAD3* gene family activity in olive has not been described yet.

The DNA methylation of gene promoter’s rich in cis-regulatory elements and TF binding sites were strictly linked to gene expression repression. It has also been shown that DNA methylation surrounding the transcription starting site (TSS) is more closely linked to transcriptional silencing [48]. The functional analysis of methylated regions in the fruit samples showed a methylation in the promoter region (−313 bp) ahead of the transcription starting site (TSS) of a putative fatty acid desaturase (FAD3) in *Ayvalik cv* (Figure 3B, Additional file S5). This enzyme is responsible for the desaturation of linoleic acid into linolenic acid in the oil biosynthesis pathway. In fact, the absence of methylation the *FAD3* gene promoter might explain the observed increase of linolenic acid content in the wild *Olea europaea ssp. europaea var. sylvestris* when compared to the cultivated *Ayvalik cv* (Table 1).

The sensory quality and aroma of EVOO depends on the biogenesis of volatile aromatic compounds, especially the fatty acid six-carbon aldehyde derivatives from linoleic and linolenic acids in the lipoxygenase pathway. Linoleate 9S-lipoxygenase (*9-LOX*; oxygen oxidoreductase) is a non-heme iron-containing enzyme that catalyzes the oxygenation of the 1,4-pentadiene sequence of polyunsaturated fatty acids to produce their corresponding hydroperoxides [36,49]. Linoleate 9S-lipoxygenase uses linoleic and linolenic acids as a substrate to form the ∆-9 hydroperoxide, which are the precursor of unsaturated six-carbon aldehydes including 2(E)-hexenal. The latter is the most prevalent constituent of olive oil aroma and responsible for the leafy green and peppery taste [50]. It has been described that the lipoxygenase activity is higher in early fruit maturation stages (15 weeks after anthesis) and decrease in advanced maturation stages (35 weeks) when the fruit are ripe and ready for harvest [51]. The methylation profiling of putative *Linoleate 9S-lipoxygenase* in both cultivated and wild olive fruits showed a methylation of the promoter of a putative linoleate 9S-lipoxygenase (−205 bp upstream) in early maturation stage for the cultivar *Ayvalik cv* (Additional file S5). Since the lipoxygenases are highly expressed in early maturation stages in the wild *Olea europaea ssp. europaea var. sylvestris* (Additional file S6), the potential silencing of one of *Ayvalik* cv lipoxygenase’s activity in early maturation due to its promoter methylation could potentially explain the observed difference in the hydroperoxidation of linoleic and linolenic acid to form their derivative volatile compounds, leading to differences in oil aroma between wild and cultivated olives. Moreover, the lower content of linoleic acid observed in the wild *Olea europaea ssp. europaea var. sylvestris* (9.08%), when compared to 10.8% in *Ayvalik cv* (Table 1), could be explained by the increased hydroperoxidation of linoleic acid as the substrate of preference to form its derivative 2(E)-hexenal. These results are consistent with previous studies on enzymatic oxidation of olive oil through the lipoxygenase pathway [51,52].

#### 3.3.2. Differential Methylation in Putative Genes Involved in Phenolic Compound Metabolism in EVOO

The phenolic composition of olive fruits and derived EVOOs is affected by many factors including cultivar, fruit development, climate conditions, and cultural practices [53,54,55]. EVOO from wild *Olea europaea ssp. europaea var. sylvestris* is characterized by a pungent taste due to the higher phenolic content when compared to the oil extracted from cultivars [6,8,9,56,57]. Several studies have focused on changes in the phenolics composition and content during fruit development in cultivated olives [58,59,60]. The total phenolic content is higher in immature drupes and gradually decreases during fruit development to reach its lowest level when fruits are ripe and ready for harvest in November [61,62,63,64]. The *GLU* expression is strictly high in the early maturation stages and might be downregulated whenever a lower availability of oleuropein occurs with fruit ripening [60]. In olives the beta-glucosidase (*OeGLU*) was also shown to have a high affinity and substrate specificity to oleuropein and plays a major role in its degradation to the aglycone form [65]. In addition, (*OeGLU*) has been demonstrated to play a central role in determining the phenolic content of olive oil [41]. In attempt to bring insight into the potential involvement of DNA methylation in the phenolic content in olive oil, we investigated the methylation status of a well characterized *OeGLU* (GenBank: AY083162.1) a beta glucosidase gene involved in phenolic compounds metabolism [66,67]. The DNA methylation profiling during fruit maturation did not manifest any differential in the methylation status of the *OeGLU* gene between the wild and cultivated olives. These results join the hypothesis from a previous study on cultivated olives *OeGLU* transcriptional activity and Oleuropein content, where the low content of oleuropein in cultivated olives was linked to differences in the regulation of enzymes involved in its biosynthesis rather than in its degradation [60]. These results raise the question about the possibility of a copy number variation in genes involved in secoiroids biosynthesis pathway that could explain the significant difference in the phenolic content between wild and cultivated olives.

### 3.4. Domestication Fingerprint on Cis-Elements Modulating Genes Activity Involved in Sensory Quality of Olive Oil

To date, domestication studies focused mainly on coding regions of high interest to the olive industry. It has been shown that the domestication had a moderate consequence on the coding sequences, and the genetic diversity was mostly functional rather than structural due to the differential in gene expression [22]. The phenotypic plasticity was also linked to the transposable element’s activation around coding genes as a result of vegetative propagation over 5000 years on millions of acres across the Mediterranean coasts [23]. DNA methylation’s role as a regulator of secondary metabolites has been largely overlooked. The comparative promoter analysis showed a weak signal of domestication on the promoter sequences of the linoleate 9S-lipoxygenase with an identity of 99% between the wild and cultivated olives. The minor presence of SNPs has not affected the *cis-regulatory* elements surrounding the methylation site (Tables S5 and S6). Altogether, these results underline the potential role of DNA methylation in the observed difference in volatile compounds responsible for the sensory quality of EVOO between wild and cultivated olives. These results are supporting the studies on *Lonicera japonica* [68], *Rorippa nasturtium var. aquaticum* [69], and *Salvia miltiorrhiza* [70], where DNA methylation was first described as a regulator of phenolic acids biosynthesis.

In contrast, the domestication through selection and vegetative propagation of olives affected the putative *FAD3* gene promoter, introducing multiple SNPs and a 12 nucleotides sequence insertion in the methylation site. This insertion led to the gain of new *cis-elements* and the loss of ancestral ones between the wild and cultivated olive trees (Table S6).

Therefore, the difference in linoleic and linolenic acids content between *Olea europaea ssp. europaea var. sylvestris* and *Ayvalik cv* are most likely due to the observed differences in *cis-elements* in the FAD3 promoter, in addition to the peroxidation of the linoleic and linolenic acids in the lipoxygenase pathway to form the six carbon volatile compounds.

## 4. Materials and Methods

### 4.1. Sample Preparation: DNA Extraction and Quantification

Wild *Olea europaea ssp. europaea var. sylvestris “karadelice”* in Turkish and *Ayvalik cv* were collected from the national germplasm collection, in Kayadibi-Izmir province Turkey. Leaves and fruits were collected in July (early maturity stage) and November (advance maturity stage) in 2014 and stored at −80 °C. The genomic DNA was isolated using a commercial kit (DNeasy Plant Maxi Kit; Qiagen Inc., Valencia, CA, USA). Following the manufacturer’s instructions. After DNA extraction, genomic DNA quantification was carried out by Qubit Fluorometer (Thermo Scientific). The DNA was fragmented by sonication using diagenode bioruptor to meet the BGI genomic DNA library preparation requirements: briefly, 10 μg/μL was used in 6 cycles of 15 s On and 90 s Off in cold water generating 300 bp fragments. The fragmented DNA was end-repaired into dA-tailed fragments using Taq polymerase (active at 72 °C for 20 min).

### 4.2. Immunoprecipitated DNA Sequencing, Data Quality Control, and Read Alignment

The immunoprecipitation assay was conducted as previously described by [71]. Briefly, 10 μg/μL of sonicated DNA was diluted in TE buffer. The diluted DNA was denatured and immediately cooled on ice; after cooling, the DNA was incubated with an Isotype IgG1 monoclonal 5mC Antibody (Epigentek Group Inc., Farmingdale, NY, USA) for 2 h at 4 °C. Dynabeads were then added to the immunoprecipitation reaction tubes and incubated for 2 h at 4 °C. After digestion, the proteinase K was added, and the reaction tube was incubated one last time for 3 h at 50 °C. At the end, the immuno-precipitated fragments were extracted with phenol/chloroform, then precipitated with NaCl (20 μL NaCl 5 M), glycogen (1 μL), and 2 volumes 100% ethanol (500 μL), and finally resuspended in TE buffer and kept at –20 °C. The library preparation and sequencing were conducted at the Beijing genome institute (BGI) using Illumina HiSeq 2000 following the manufacturer guidelines and generating 63 to 65 M reads of raw data per sample with a Q20 between 97.64 and 98.80 (Table S1). The raw reads were subject to quality control using FASTQC v0.11.9, and the statistics of clean data after QC are available in Supplemental Table S2. After the quality control, reads were trimmed using Trimomatic V0.39 [72] to remove adapters and filter out the reads when N content greater than 5%. Low-quality raw reads were filtered before running quality control. The clean reads were then mapped to the wild *Olea europaea* *ssp*. *europaea var. sylvestris* genome sequence v1 [11] using BWA-0.7.17 [73] and resulting in a uniquely read mapping average rate of 94%. (Table S3). Samtools v1.10 [74] were used to sort, filter, index, and run statistics on the BAM files. Next, uniquely mapped read density distribution in chromosomes was visualized in a Manhattan plot using Circos v0.69.9 [75].

### 4.3. Methylated Regions Calling, Annotation, and Functional Analysis

To detect the methylated region in wild and cultivated olives, peaks related to genes, their count, width, significance, and distribution throughout genomic features including transcription starting site (TSS), transcription termination site (TTS), exons, and introns were called using MACS v2.2.7 software [76] with the following parameters (threshold -g 1.2e8-p 1e-5 -m 10,100). The width of the peak indicates the length of the DNA sequence to which the methylation-specific antibody protein binds. The significance of the peak (*p*-value) is an indication of the degree of confidence. The fold enrichment can also be called signal value, indicating a digital display of the peak signal during the peak calling. Larger values correspond to the number of enriched reads on the peak. Homer v4.10 annotate peak Python program [77] was used to annotate the genes associated with methylated regions in both cultivated and wild olive. HOMER first determines the distance to the nearest TSS and assigns the peak to that gene. Second, it determines the genomic annotation of the region occupied by the center of the peak using a gene transfer format (GTF) to retrieve structural annotation. In addition to associating peaks with nearby genes, HOMER was used to perform gene ontology and KEGG pathway analysis. The bar charts for different gene ontology terms (molecular function, cellular component, and biological process) and the scatter plots for the KEGG enrichment analysis were dressed using GO plot v1.0.2 [78]. To emphasize the role of DNA methylation in modulating the metabolic activity, differentially methylated gene IDs were extracted from the most enriched pathways in fatty acids and phenolic compounds metabolism. The gene IDs were mapped against the wild *Olea europaea var. sylvestris* annotation database hosted in the online resource for community annotation of eukaryotes [79] for further functional annotation. To explain the DNA methylation dynamic across tissues and maturation stages, DNA methyltransferases, and demethylases IDs were extracted from the Olea europaea var sylvestris annotation database and mapped against the published *Olea europaea ssp. europaea var. sylvestris* (July/November) differential gene expression matrix (Additional file S6).

### 4.4. Comparative Promoter Analysis

To explore the cis-regulatory elements of methylated gene between *Ayvalik cv* and wild *Karadelice*, a 1 kb promoter upstream the transcription starting site of two methylated genes: linoleate 9S lipoxugenase (*9-LOX*) and fatty acid desaturase (*FAD3*) were analyzed. The selected 1 Kb promoter sequences were extracted from the online resource for community annotation of eukaryotes genome browser using gene coordinates [79]. The three promoter sequences from the *Olea europaea ssp. europaea var. sylvestris* reference genome [11] were mapped against the *Ayvalik* assembly scaffolds provided by olive genome consortium using standalone BLASTN 2.6.0+ [80]. The homologous promoter sequences were subject to a *cis-element* analysis using Genomatix MatInspector v8.4.1 [81]. To avoid any discrepancies in methylation site calling, cis-elements screening windows were extended to the 24 bp flanking the predicted peak summit.

### 4.5. Metabolic Analysis, Fatty Acid and Total Polyphenol Profiling

The mono cultivar certified commercial olive oil sample from *Olea europaea ssp. europaea var. sylvestris* “*karadelice*” were kindly provided by Egeden Dogal Tarim Urunleri Ltd., Mugla, Turkey. To determine the fatty acid and total phenolic content, the gas chromatography flame ionization detector (GC-FID) and high-performance liquid chromatography (HPLC-UV) as described by [82,83] were performed in TARIM VE ORMAN BAKANLIĞI AYTB Hizmetleri Laboratory in turkey. The Nuclear Magnetic Resonance (NMR) analysis for detailed secoiridoids compounds was conducted in the laboratory of Natural Products Chemistry, Faculty of Pharmacy at the National and Kapodistrian University of Athens following the method described by [84]. Since the olive oil from *Ayvalik cv* is well and extensively characterized we used mean value from the previous studies [4,5].

## 5. Conclusions

The current study provides a base to understanding the DNA methylation landscape evolution throughout domestication of olive trees in the Aegean region of Turkey. DNA methylation could fill the gap in explaining the unassigned genetic variation responsible for the phenotypic plasticity in *Olea europaea* species. The genome-wide DNA methylation profiling of cultivated and wild olives from Turkey described the genome wide methylation status in olive fruit during ripening. The study also describes the potential role of DNA methylation in volatile aromatic compounds metabolism in the LOX pathway. The study provides a platform for epigenetic markers discovery, that could be used for early screening in olive trees breeding. It is recommended to carry out additional studies with larger samples of wild and cultivated olives from eastern and western Mediterranean regions, in addition to combining metabolomic, transcriptomic, and epigenomic data to elucidate the complexity of the biology and the evolution of this endemic tree.

### Remarks and Future Perspectives

Exploring epigenetic diversity for plant breeding was widely studied in model plants and horticulture, but still rudimentary in sessile and long lived agro-forest trees [85,86]. This study is a gateway transition to explore epigenetics as a tool in agroforestry. Understanding the role of epigenetic variability in forest species and how it may contribute to their rapid adaptation to changing environments should be of high interest, especially with the current drastic climate change.

In the midst of the current climate change debate, food security has become of extreme concern to the food and agriculture sectors. Increasing crop production represents a great challenge in many countries. The food and agriculture organization (FAO), in partnership with local entities launched a variety of national programs for crop micro propagation and tissue culture of fruit trees in arid, semiarid, and tropical regions for conservation and restoration of damaged forests, groves, and orchards. For instance, to restore the date palm groves ravaged by *Bayoud Fusarium wilt* disease, efforts to produce clones resistant to the disease have been established in Saudi Arabia, the UAE, Morocco, and other North African and middle eastern countries since the 1980s. At this time, palm groves are still suffering from the lack of uniformity in established clones. In the southern coast of Europe, especially Italy, in an effort to contain the spread of *Xylella fastidiosa* in olive groves in the eastern Mediterranean and North Africa, the FAO is encouraging countries importing explants to produce their own through micropropagation and tissue culture. In Morocco, multiple efforts and resources are being deployed by the Kingdom in partnership with the FAO to restore the wild Moroccan Argan (*Argania spinosa*) tree groves in the Atlas Mountain valley, because of its high socioeconomic importance.

To avoid scenarios similar to the oil palm catastrophe in Indonesia [87], national programs on micro propagation and tissue culture should also take into consideration the recommendation from the scientific community. In addition to the socioeconomic recommendation, studying micropropagation effects on genome rearrangement and DNA methylation dynamics should be taken into utmost consideration.

## Figures and Tables

**Table 1:** Mean values of the major quantified fatty acids in (%), phenolic compounds (mg/kg). Values for *Ayvalik cv* EVOO represent the mean value of three replicate analyses data from [4,5]. Values for *Olea europaea ssp. europaea var. sylvestris* EVOO from the GC-FID HPLC-UV, and NMR represent the average of two replicates analysis. Abbreviations: ND not detected, diAOle agl dialdehyde form of oleuropein aglycone, AOle agl aldehydic form of oleuropein aglycone, Alig agl aldehydic form of ligstroside aglycon.

Compound	C18:1	C18:2	C183	Oleocanthal	Oleacein	Aole agl	diAOle agl	Alig agl	diALig agl	Total Polyphenol
***Ayvalik cv* EVOO**	69.3	10.8	0.7	4.9	4.2	3.1	0.8	2.7	ND	227
**wild EVOO**	71.07	9.08	0.98	74	76	216	462	81	521	1034

## Supplementary Materials

The following are available online at https://www.mdpi.com/article/10.3390/plants10071405/s1, Additional file S1: promoter alignment between wild and cultivated olives. Additional file S2: Leaves methyltransferases differential gene expression between July and November. Additional file S3: fruit methyltransferases differential gene expression between July and November. Additional file S4: fruit demethylases differential gene expression between July and November. Additional file S5: fruit differential methylation annotation between wild and cultivated olives. Additional file S6: wild oleaster fruit DGE between July and November. Additional file S7: phenolic compound analysis for Oleaster. Figure S1: Differential methylation between leaves and fruit in wild and cultivated olive trees, Figure S2: Differential methylated profile between the wild oleaster (Delice) and cultivated Ayvalik within the 23 olive chromosomes, Figure S3: Differential methylated region in fruits and leaves between July and November in wild and cultivated olive trees, Figure S4: Differential methylated region in fruits and leaves between wild and cultivated olive trees in different maturation stages, Figure S5: Heatmap of leaves DNA methyltransferases differential genes expression (DGE) between July and November (DGE data published in Turgay et al., 2017), Figure S6: Heatmap of fruit DNA methyltransferases differential genes expression between July and November (DGE data published in Turgay et al., 2017), Figure S7: Heatmap of fruits DNA demethylases differential genes expression between July and November, Table S1: Raw data statistic information, Table S2: Statistics of clean data after QC and trimming, Table S3: Statistics of reads alignments, Table S4: Summary of gene ontology and KEGG enrichment of DMR between wild Delice and cultivated Ayvalik for fruits and leaves in different fruit maturation phases (A: *Ayvalik*; D: *Delice*; J: July; N: November; L: leaf; F: fruit), Table S5: Identified cis-elements at the lipoxygenase 1 (Oeu003662.1) involved in the methylated peak/site, Table S6: Identified cis-elements at the methylation site for the fatty acid desaturase-3 (Oeu032520) promoter.
