# Supplementary material for: Genome Wide MeDIP-Seq Profiling of Wild and Cultivated Olives Trees Suggests DNA Methylation Fingerprint on the Sensory Quality of Olive Oil
Source: Plants (Basel). 2021 Jul 9;10(7):1405. doi: 10.3390/plants10071405 (PMC8309279; doi:10.3390/plants10071405)
Supplement: Supplementary file 1 [file plants-10-01405-s001.zip › Additional file 7. phenolic compound analysis for Oleaster.pptx]

## Slide 1
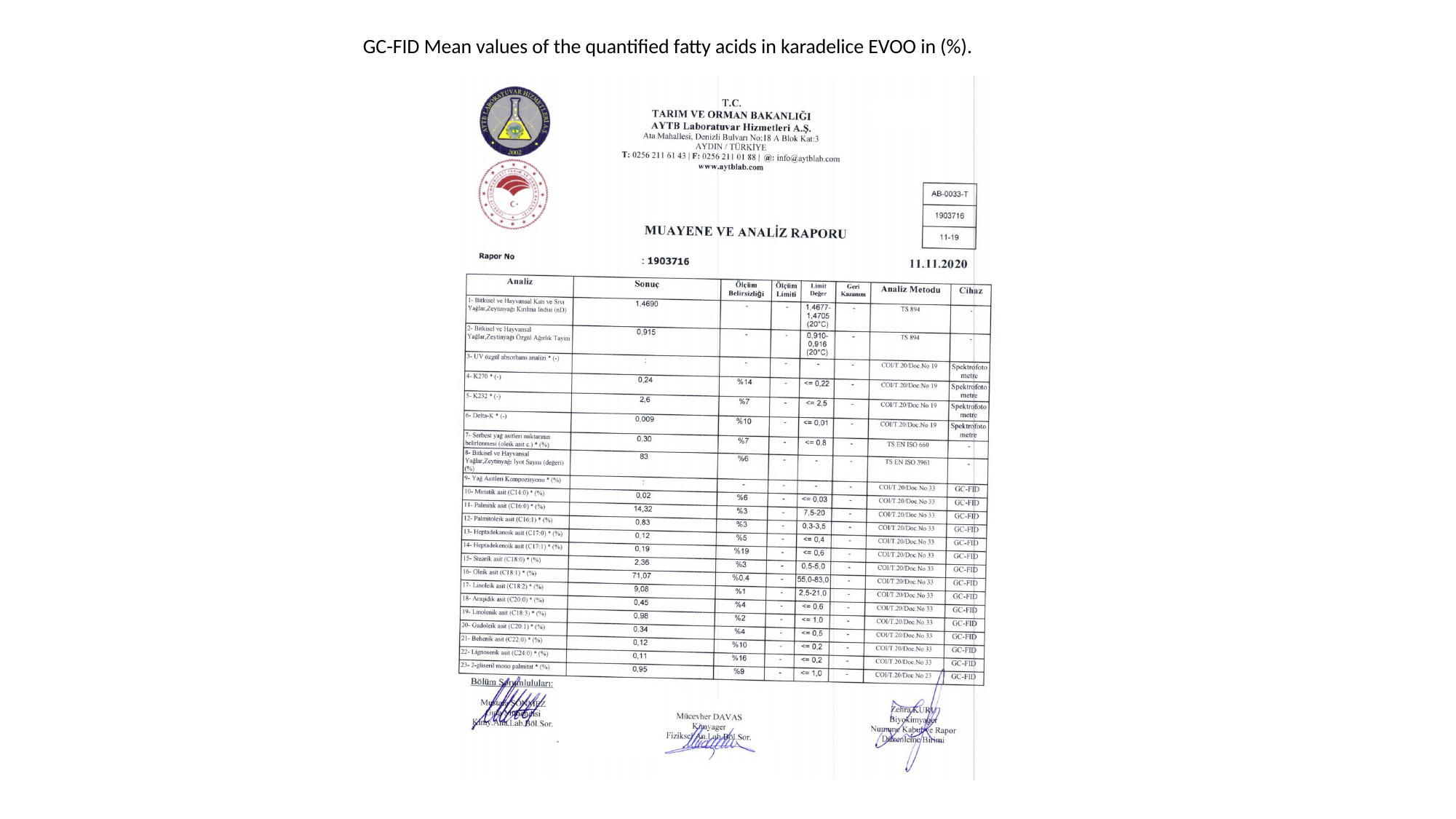

# GC-FID Mean values of the quantified fatty acids in karadelice EVOO in (%).

## Slide 2
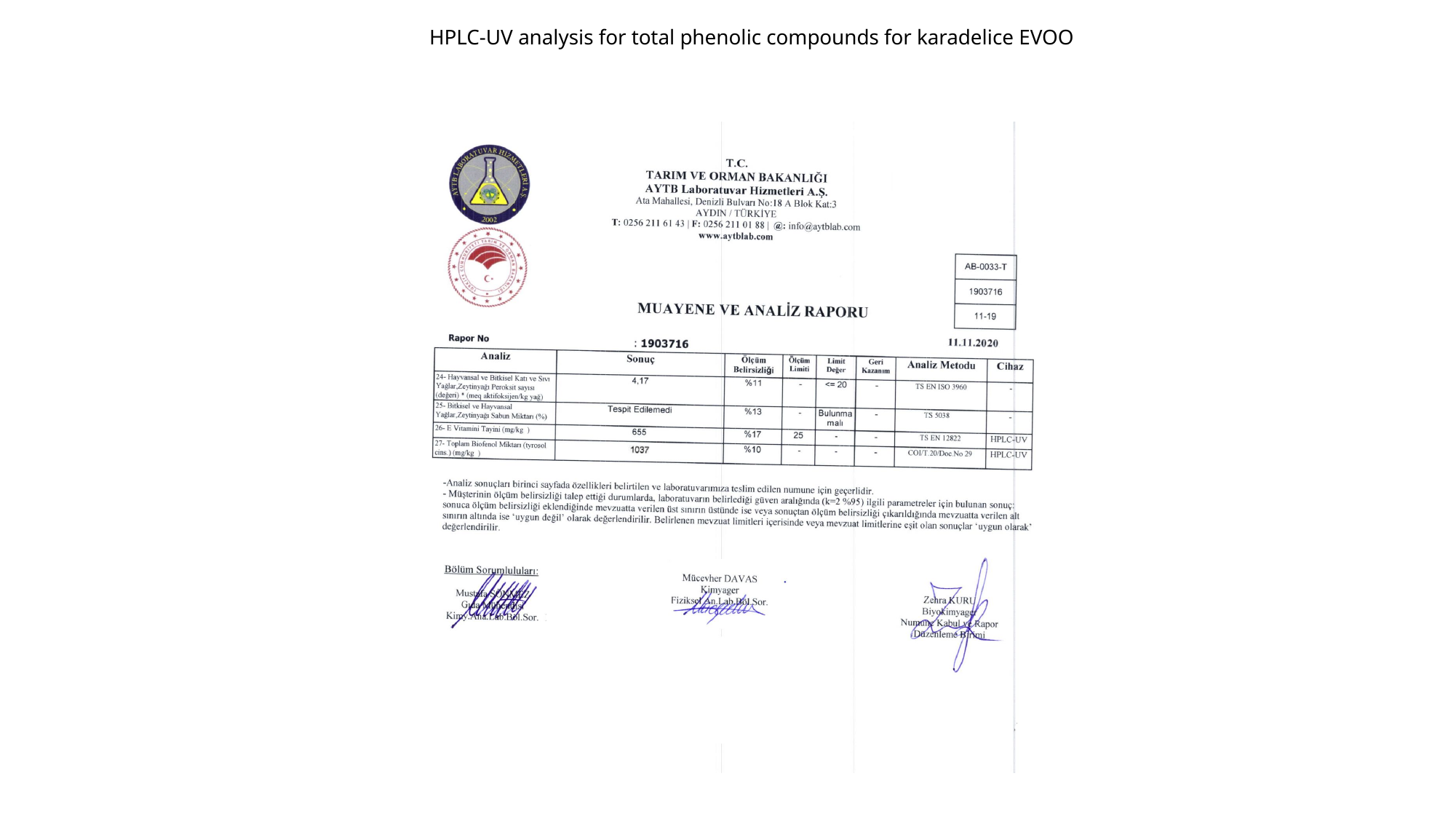

# HPLC-UV analysis for total phenolic compounds for karadelice EVOO

## Slide 3
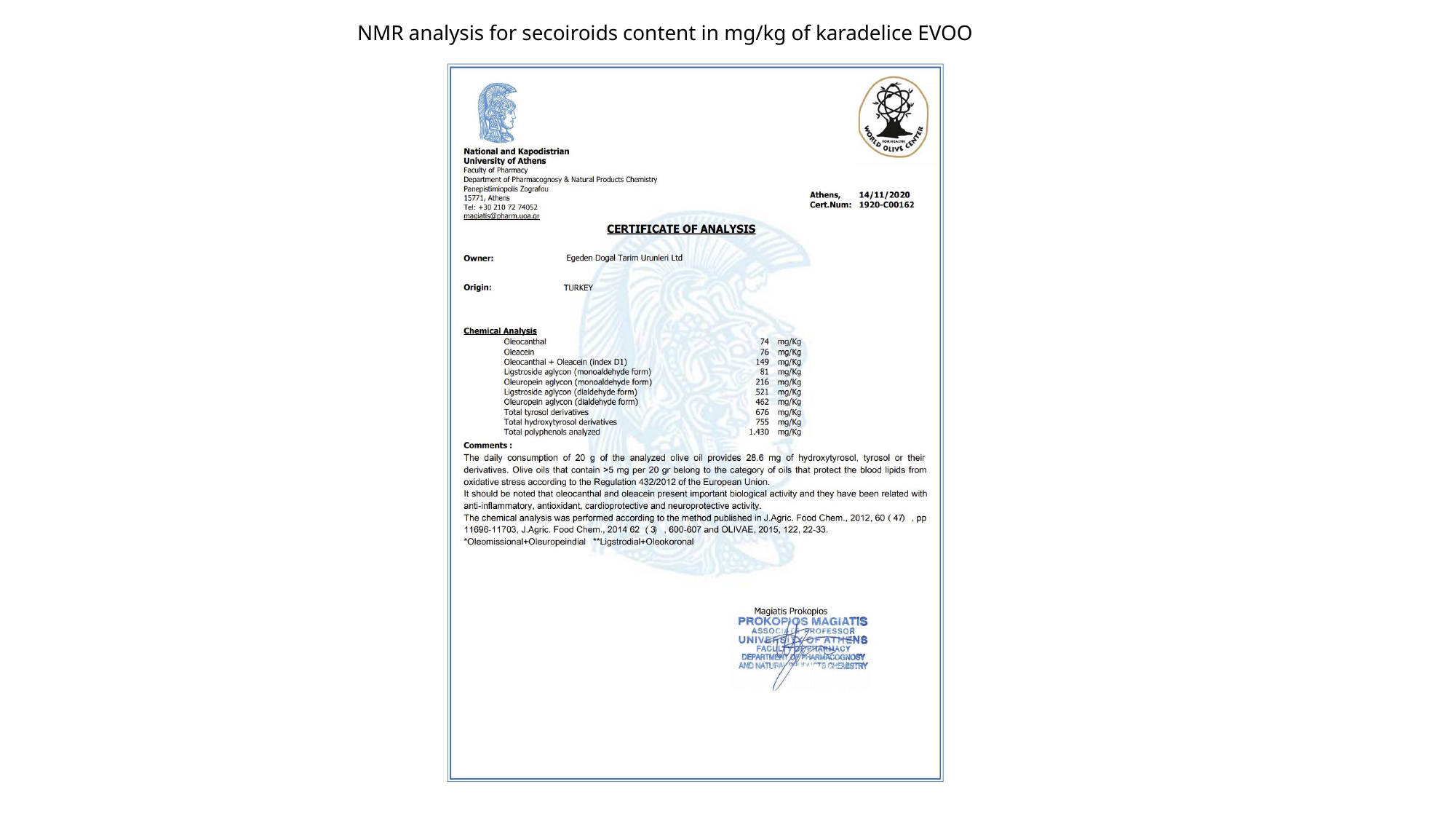

# NMR analysis for secoiroids content in mg/kg of karadelice EVOO
