# Supplementary material for: Genome Wide MeDIP-Seq Profiling of Wild and Cultivated Olives Trees Suggests DNA Methylation Fingerprint on the Sensory Quality of Olive Oil
Source: Plants (Basel). 2021 Jul 9;10(7):1405. doi: 10.3390/plants10071405 (PMC8309279; doi:10.3390/plants10071405)
Supplement: Supplementary file 1 [file plants-10-01405-s001.zip › supplementary figures and tables Manuscript ID plants-1226505.pptx]

## Slide 1
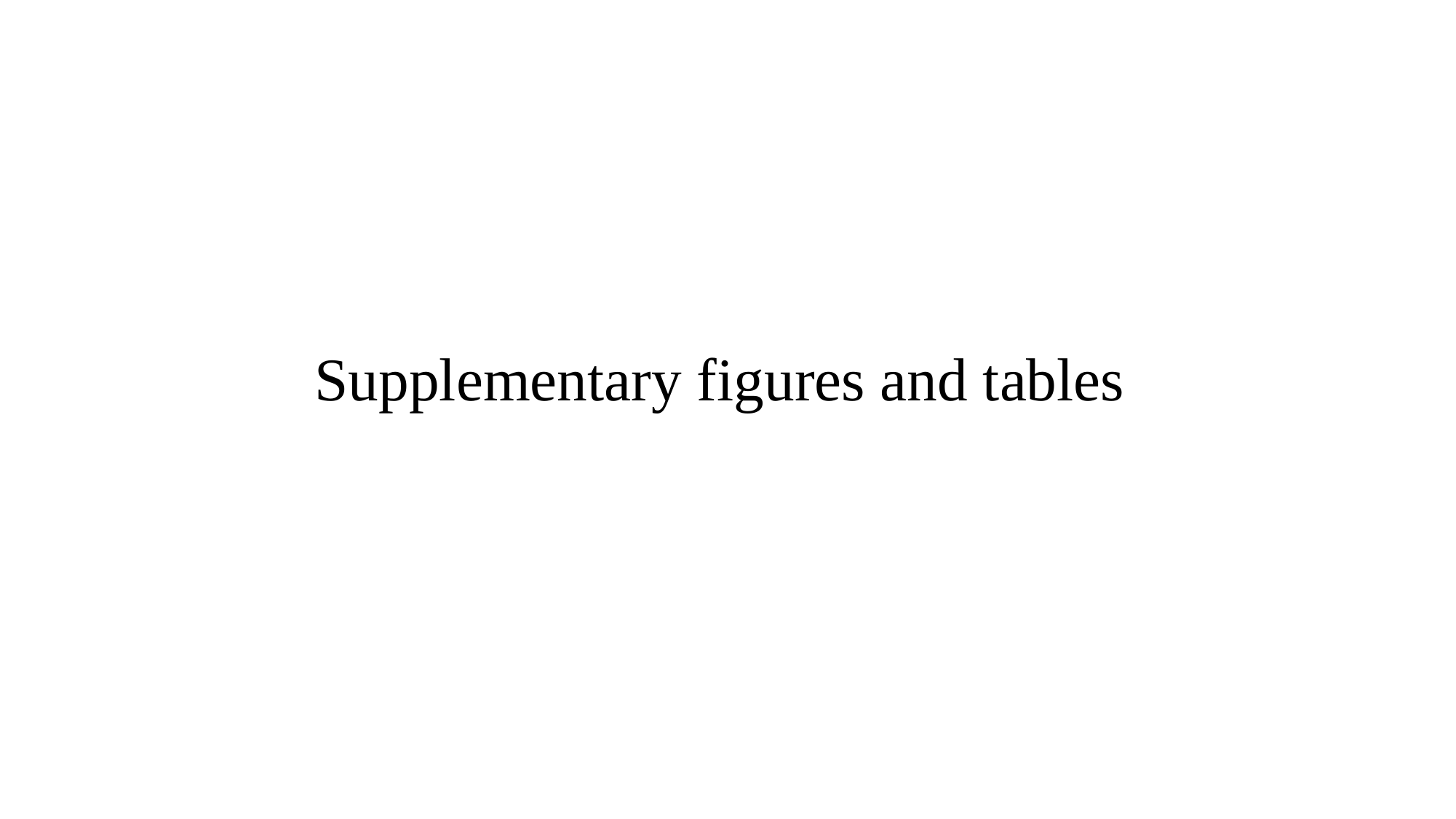

# Supplementary figures and tables

## Slide 2
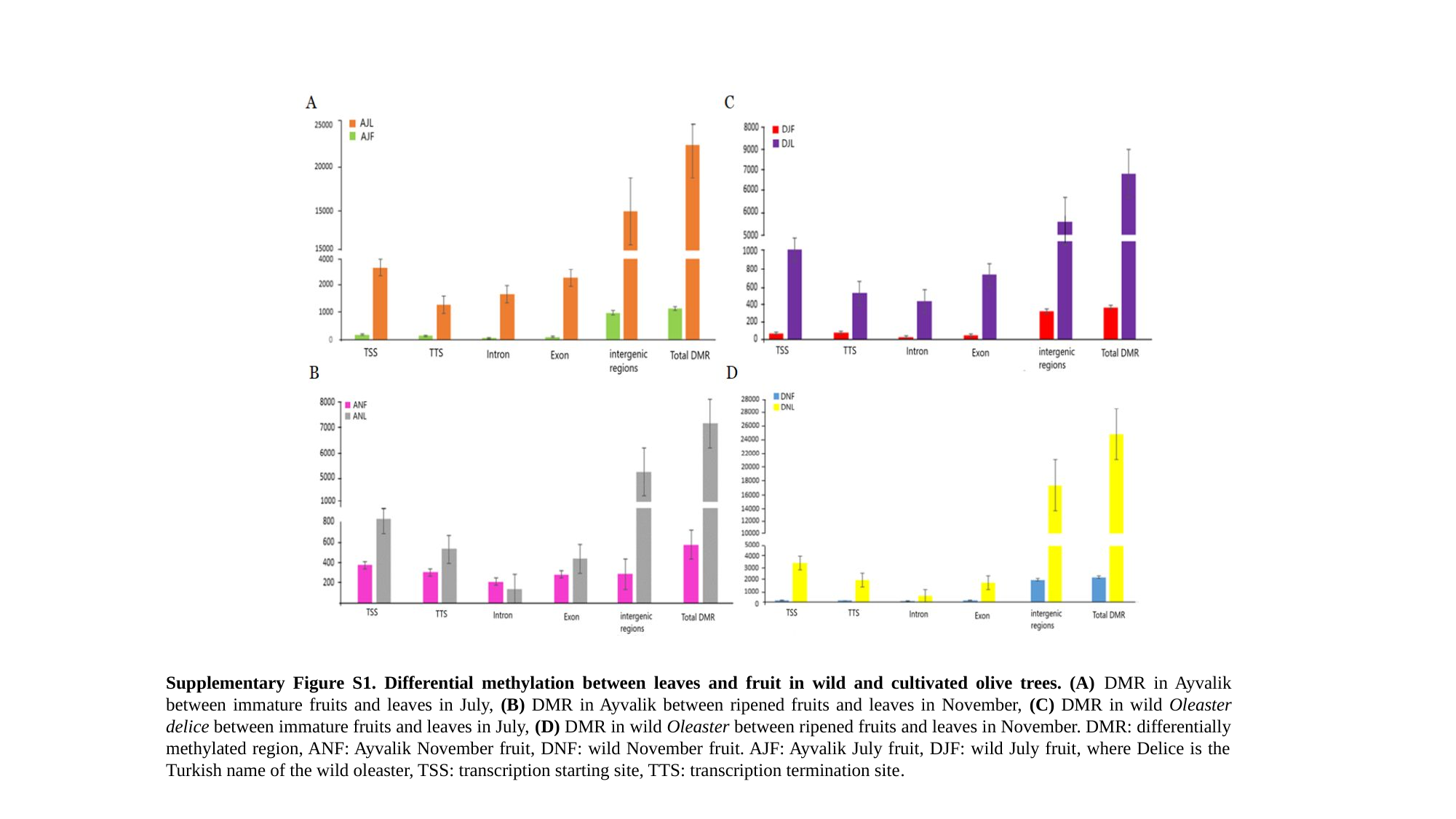

Supplementary Figure S1. Differential methylation between leaves and fruit in wild and cultivated olive trees. (A) DMR in Ayvalik between immature fruits and leaves in July, (B) DMR in Ayvalik between ripened fruits and leaves in November, (C) DMR in wild Oleaster delice between immature fruits and leaves in July, (D) DMR in wild Oleaster between ripened fruits and leaves in November. DMR: differentially methylated region, ANF: Ayvalik November fruit, DNF: wild November fruit. AJF: Ayvalik July fruit, DJF: wild July fruit, where Delice is the Turkish name of the wild oleaster, TSS: transcription starting site, TTS: transcription termination site.

## Slide 3
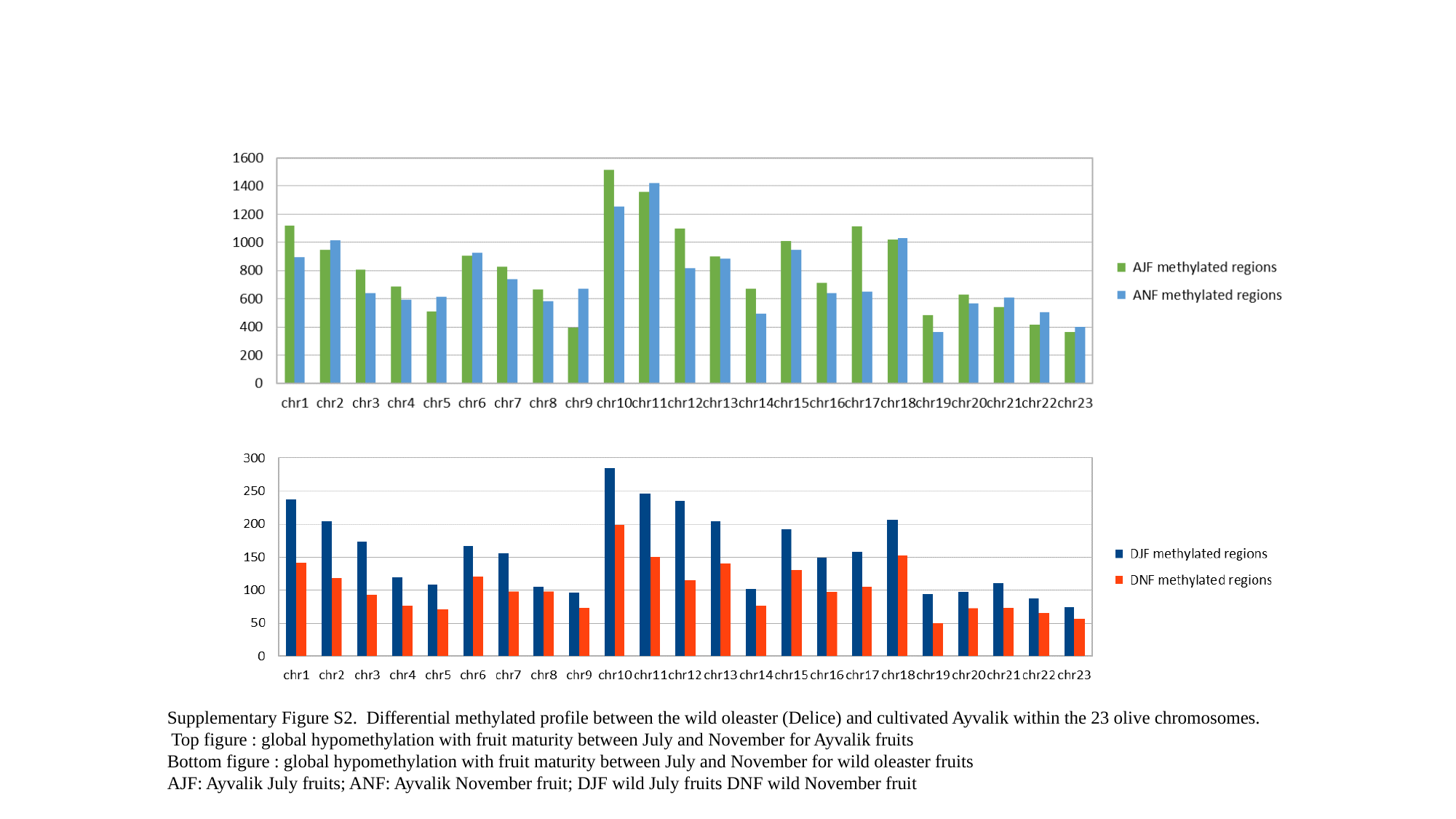

# Supplementary Figure S2. Differential methylated profile between the wild oleaster (Delice) and cultivated Ayvalik within the 23 olive chromosomes. Top figure : global hypomethylation with fruit maturity between July and November for Ayvalik fruits Bottom figure : global hypomethylation with fruit maturity between July and November for wild oleaster fruits AJF: Ayvalik July fruits; ANF: Ayvalik November fruit; DJF wild July fruits DNF wild November fruit

## Slide 4
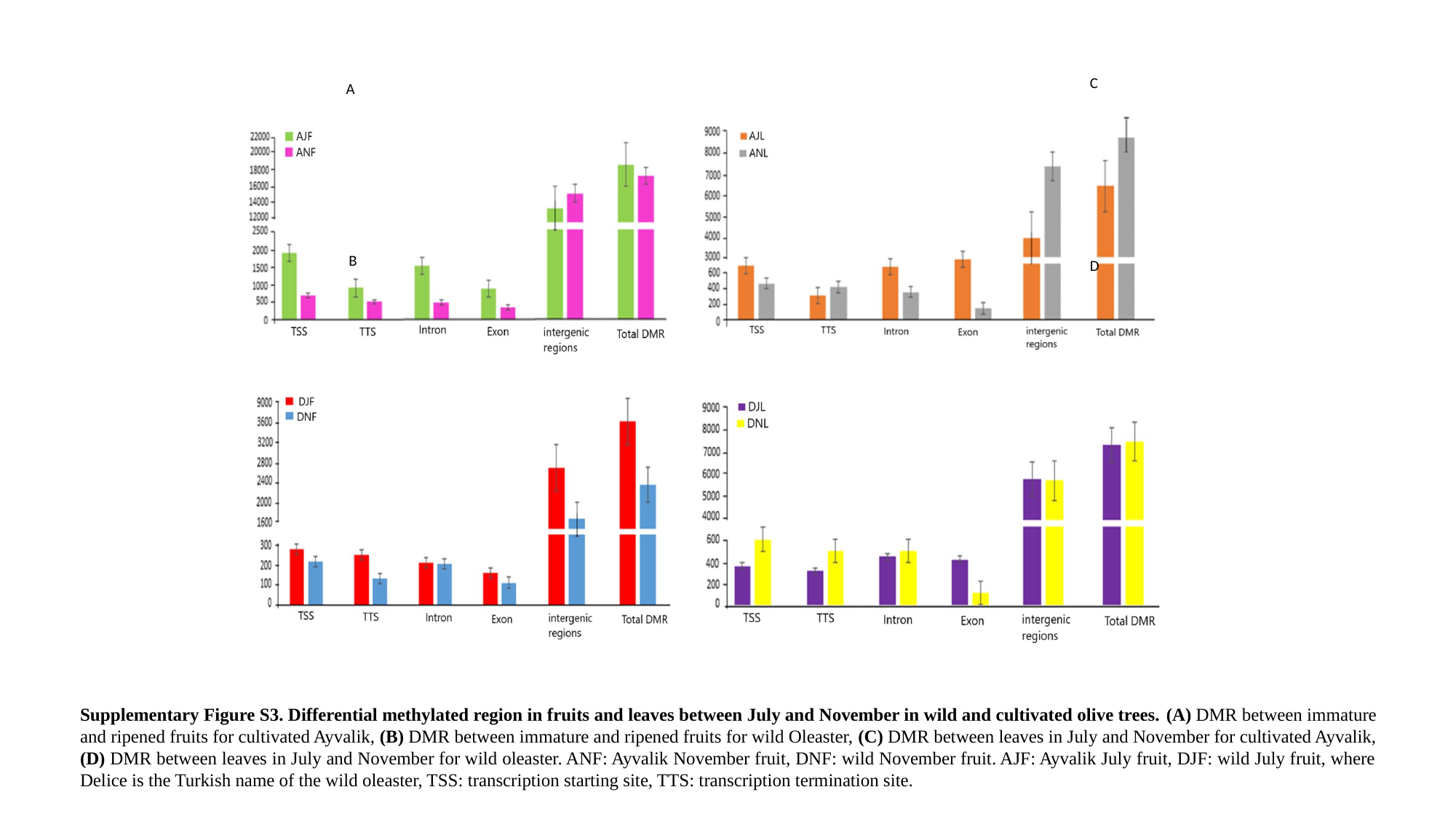

C
A
B
D
Supplementary Figure S3. Differential methylated region in fruits and leaves between July and November in wild and cultivated olive trees. (A) DMR between immature and ripened fruits for cultivated Ayvalik, (B) DMR between immature and ripened fruits for wild Oleaster, (C) DMR between leaves in July and November for cultivated Ayvalik, (D) DMR between leaves in July and November for wild oleaster. ANF: Ayvalik November fruit, DNF: wild November fruit. AJF: Ayvalik July fruit, DJF: wild July fruit, where Delice is the Turkish name of the wild oleaster, TSS: transcription starting site, TTS: transcription termination site.

## Slide 5
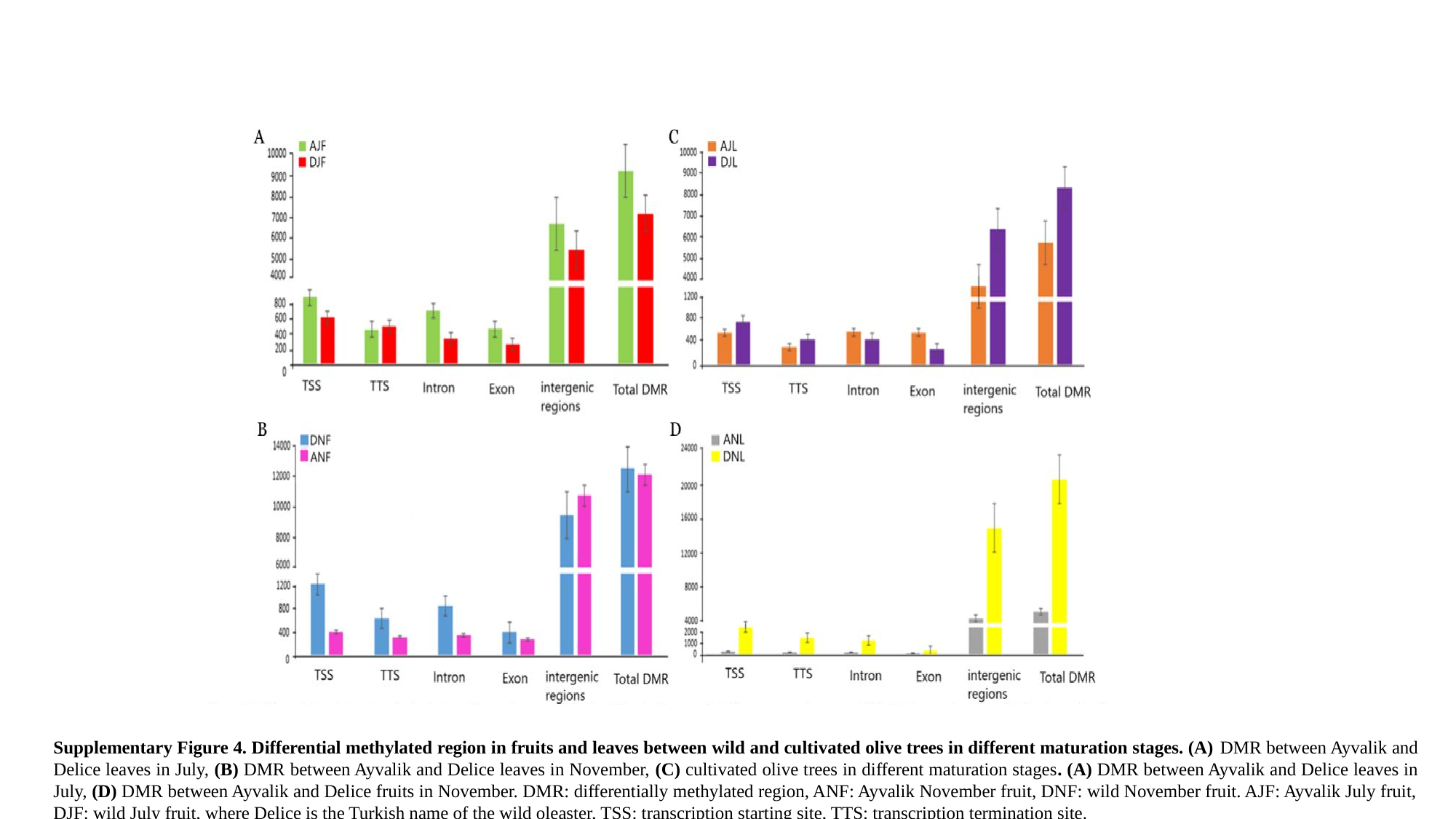

Supplementary Figure 4. Differential methylated region in fruits and leaves between wild and cultivated olive trees in different maturation stages. (A) DMR between Ayvalik and Delice leaves in July, (B) DMR between Ayvalik and Delice leaves in November, (C) cultivated olive trees in different maturation stages. (A) DMR between Ayvalik and Delice leaves in July, (D) DMR between Ayvalik and Delice fruits in November. DMR: differentially methylated region, ANF: Ayvalik November fruit, DNF: wild November fruit. AJF: Ayvalik July fruit, DJF: wild July fruit, where Delice is the Turkish name of the wild oleaster, TSS: transcription starting site, TTS: transcription termination site.

## Slide 6
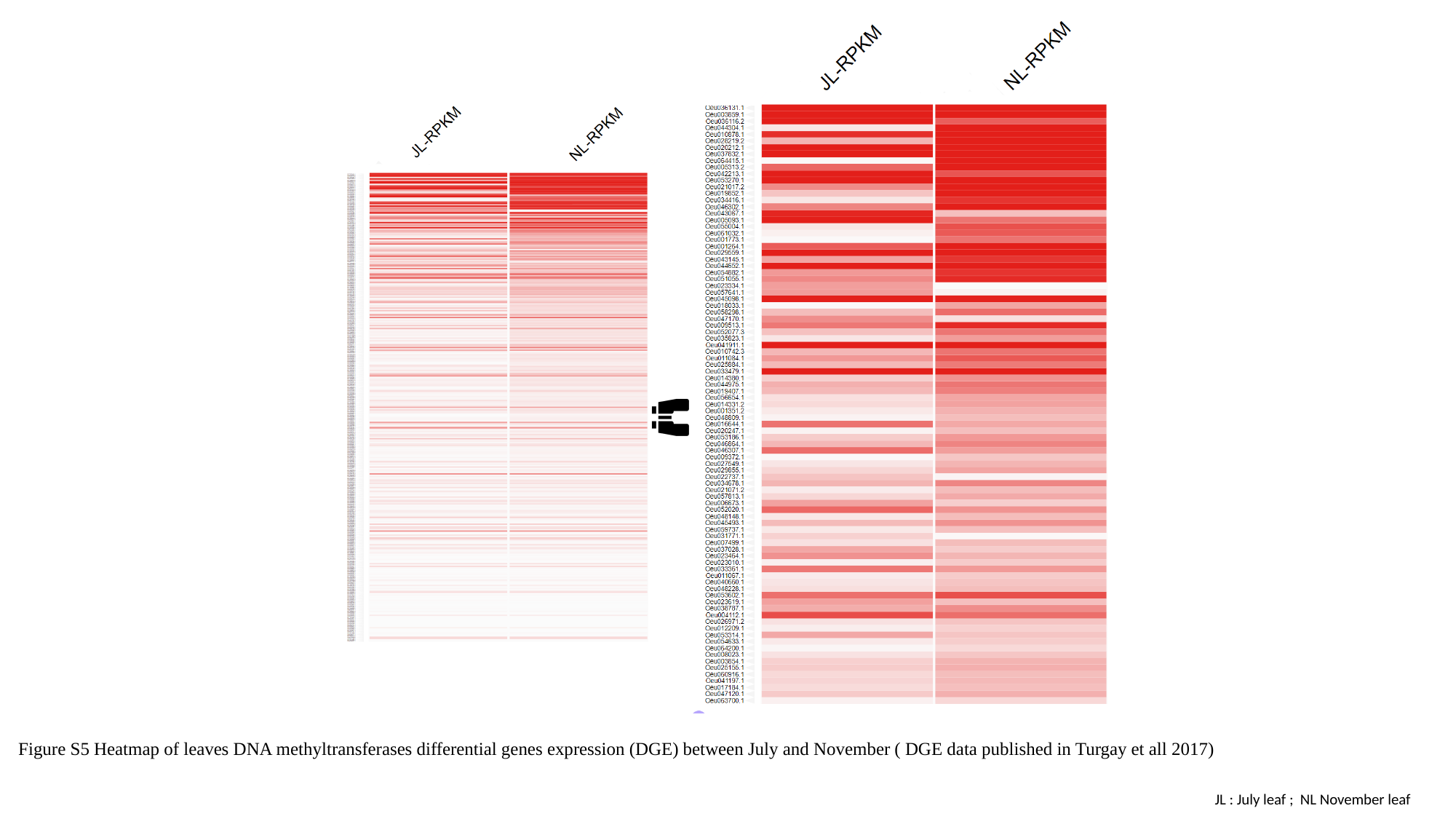

# Figure S5 Heatmap of leaves DNA methyltransferases differential genes expression (DGE) between July and November ( DGE data published in Turgay et all 2017)
JL : July leaf ; NL November leaf

## Slide 7
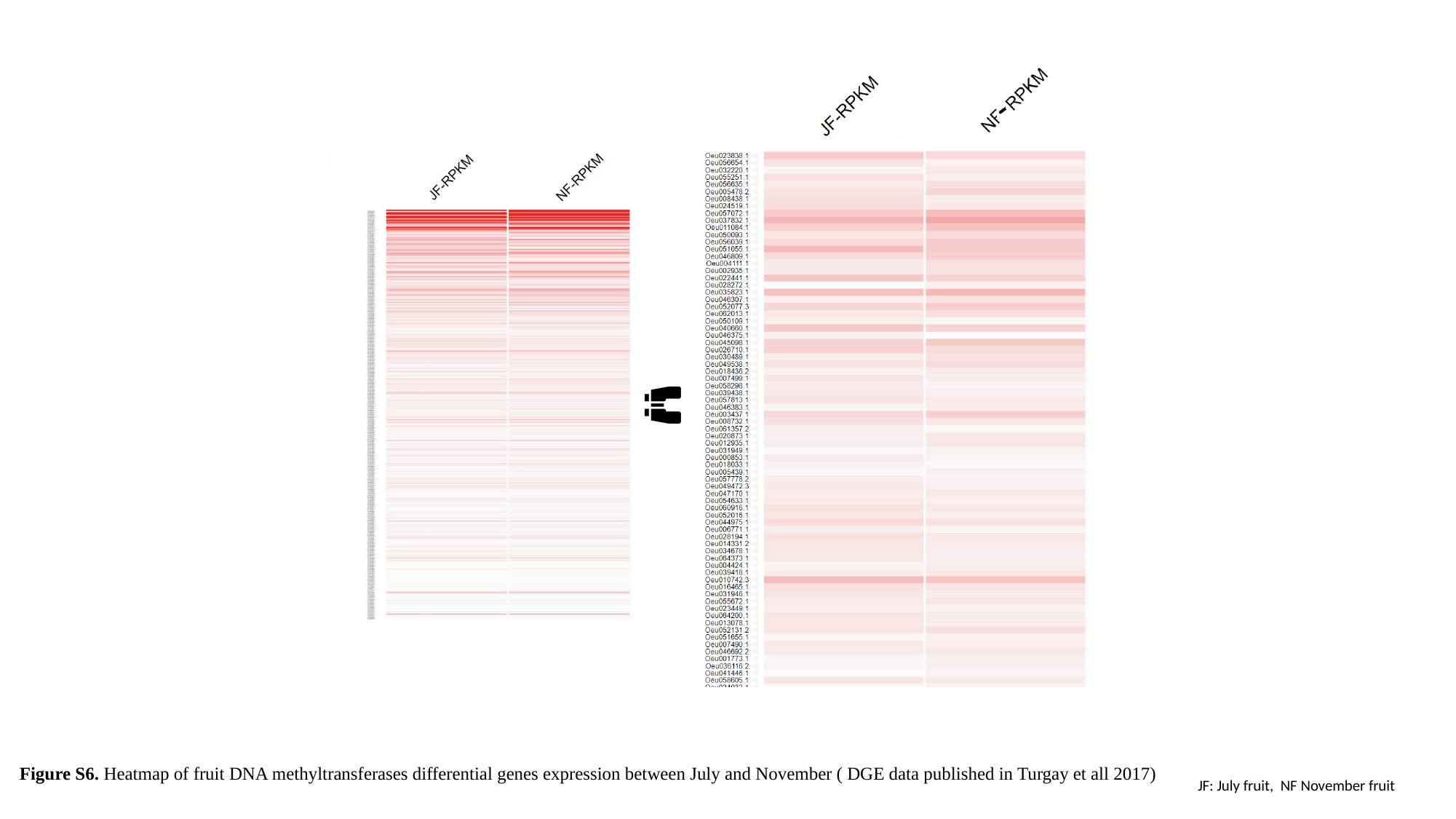

# Figure S6. Heatmap of fruit DNA methyltransferases differential genes expression between July and November ( DGE data published in Turgay et all 2017)
JF: July fruit, NF November fruit

## Slide 8
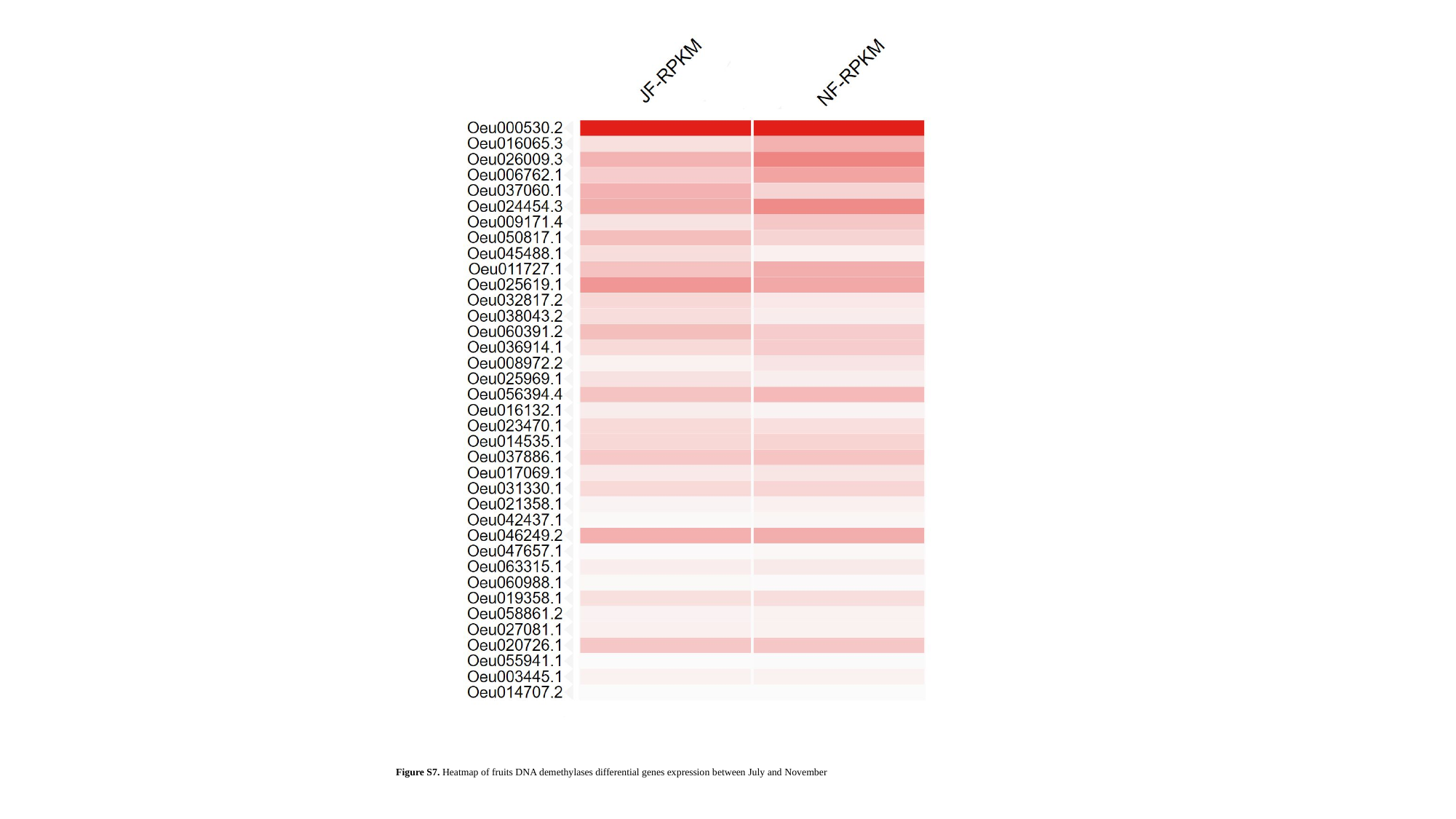

# Figure S7. Heatmap of fruits DNA demethylases differential genes expression between July and November

## Slide 9
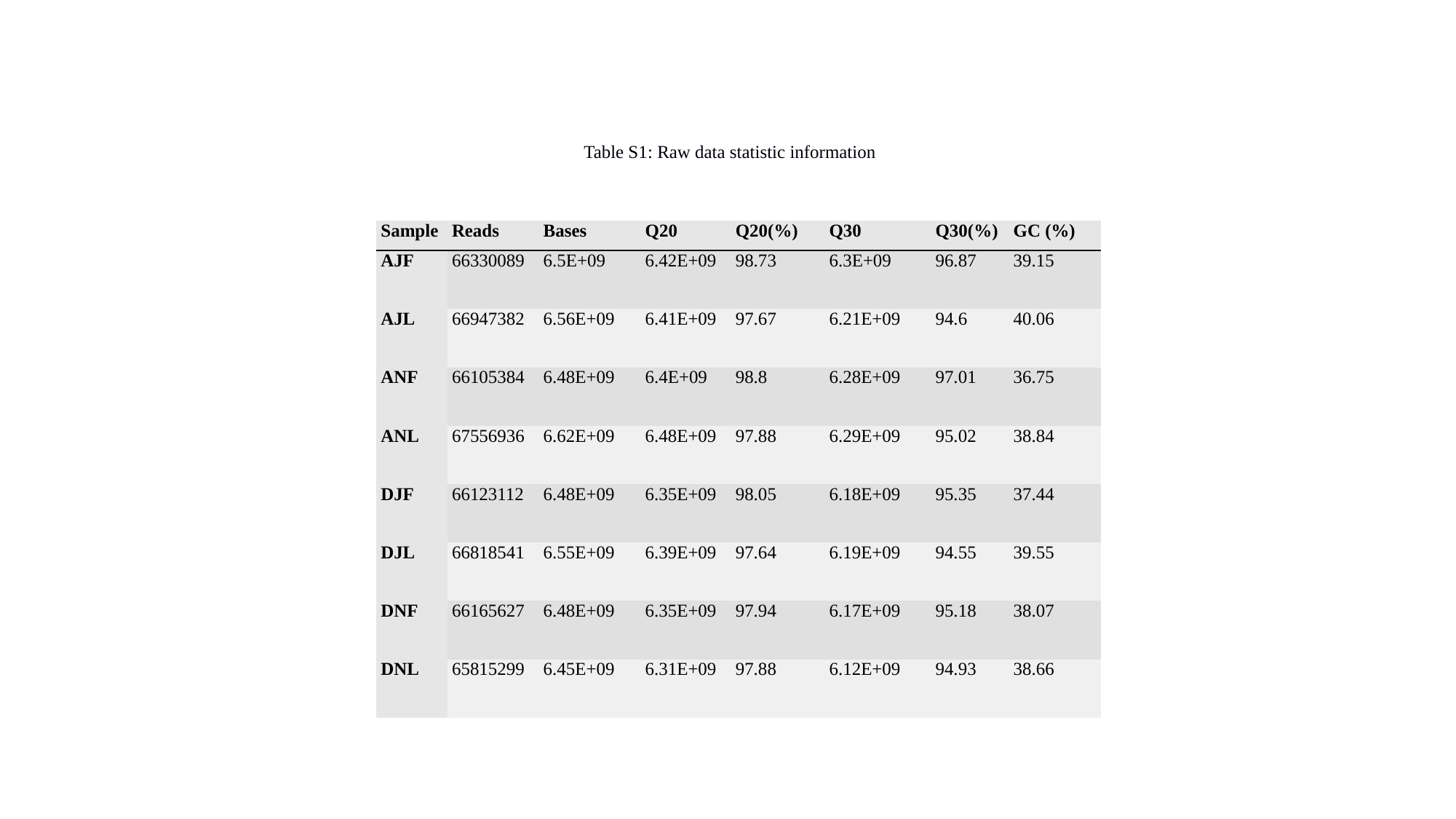

# Table S1: Raw data statistic information
| Sample | Reads | Bases | Q20 | Q20(%) | Q30 | Q30(%) | GC (%) |
| --- | --- | --- | --- | --- | --- | --- | --- |
| AJF | 66330089 | 6.5E+09 | 6.42E+09 | 98.73 | 6.3E+09 | 96.87 | 39.15 |
| AJL | 66947382 | 6.56E+09 | 6.41E+09 | 97.67 | 6.21E+09 | 94.6 | 40.06 |
| ANF | 66105384 | 6.48E+09 | 6.4E+09 | 98.8 | 6.28E+09 | 97.01 | 36.75 |
| ANL | 67556936 | 6.62E+09 | 6.48E+09 | 97.88 | 6.29E+09 | 95.02 | 38.84 |
| DJF | 66123112 | 6.48E+09 | 6.35E+09 | 98.05 | 6.18E+09 | 95.35 | 37.44 |
| DJL | 66818541 | 6.55E+09 | 6.39E+09 | 97.64 | 6.19E+09 | 94.55 | 39.55 |
| DNF | 66165627 | 6.48E+09 | 6.35E+09 | 97.94 | 6.17E+09 | 95.18 | 38.07 |
| DNL | 65815299 | 6.45E+09 | 6.31E+09 | 97.88 | 6.12E+09 | 94.93 | 38.66 |
| Sample | Reads | Bases | Q20 | Q20(%) | Q30 | Q30(%) | GC (%) |
| --- | --- | --- | --- | --- | --- | --- | --- |
| AJF | 66330089 | 6.5E+09 | 6.42E+09 | 98.73 | 6.3E+09 | 96.87 | 39.15 |
| AJL | 66947382 | 6.56E+09 | 6.41E+09 | 97.67 | 6.21E+09 | 94.6 | 40.06 |
| ANF | 66105384 | 6.48E+09 | 6.4E+09 | 98.8 | 6.28E+09 | 97.01 | 36.75 |
| ANL | 67556936 | 6.62E+09 | 6.48E+09 | 97.88 | 6.29E+09 | 95.02 | 38.84 |
| DJF | 66123112 | 6.48E+09 | 6.35E+09 | 98.05 | 6.18E+09 | 95.35 | 37.44 |
| DJL | 66818541 | 6.55E+09 | 6.39E+09 | 97.64 | 6.19E+09 | 94.55 | 39.55 |
| DNF | 66165627 | 6.48E+09 | 6.35E+09 | 97.94 | 6.17E+09 | 95.18 | 38.07 |
| DNL | 65815299 | 6.45E+09 | 6.31E+09 | 97.88 | 6.12E+09 | 94.93 | 38.66 |

## Slide 10
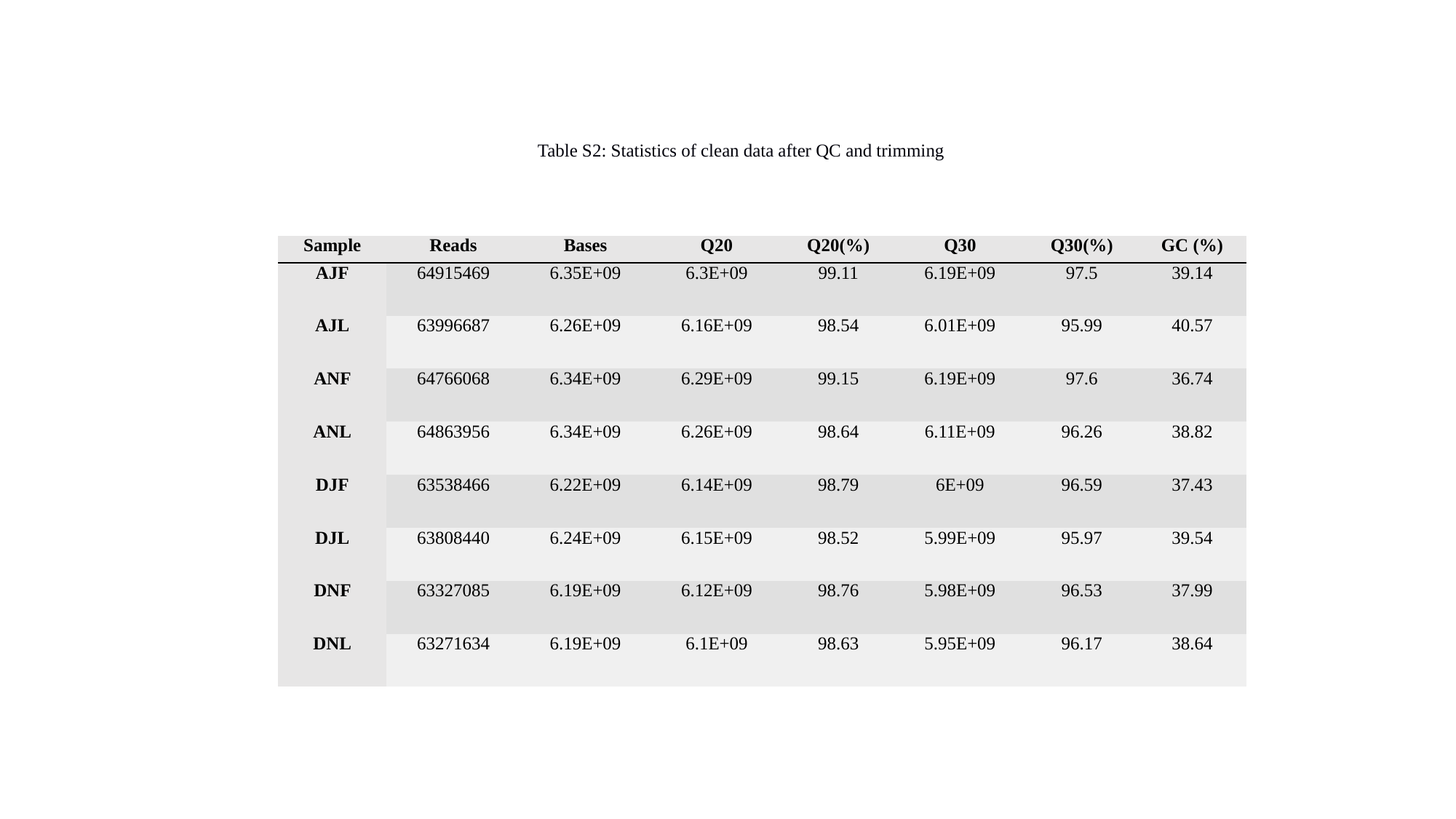

# Table S2: Statistics of clean data after QC and trimming
| Sample | Reads | Bases | Q20 | Q20(%) | Q30 | Q30(%) | GC (%) |
| --- | --- | --- | --- | --- | --- | --- | --- |
| AJF | 64915469 | 6.35E+09 | 6.3E+09 | 99.11 | 6.19E+09 | 97.5 | 39.14 |
| AJL | 63996687 | 6.26E+09 | 6.16E+09 | 98.54 | 6.01E+09 | 95.99 | 40.57 |
| ANF | 64766068 | 6.34E+09 | 6.29E+09 | 99.15 | 6.19E+09 | 97.6 | 36.74 |
| ANL | 64863956 | 6.34E+09 | 6.26E+09 | 98.64 | 6.11E+09 | 96.26 | 38.82 |
| DJF | 63538466 | 6.22E+09 | 6.14E+09 | 98.79 | 6E+09 | 96.59 | 37.43 |
| DJL | 63808440 | 6.24E+09 | 6.15E+09 | 98.52 | 5.99E+09 | 95.97 | 39.54 |
| DNF | 63327085 | 6.19E+09 | 6.12E+09 | 98.76 | 5.98E+09 | 96.53 | 37.99 |
| DNL | 63271634 | 6.19E+09 | 6.1E+09 | 98.63 | 5.95E+09 | 96.17 | 38.64 |

## Slide 11
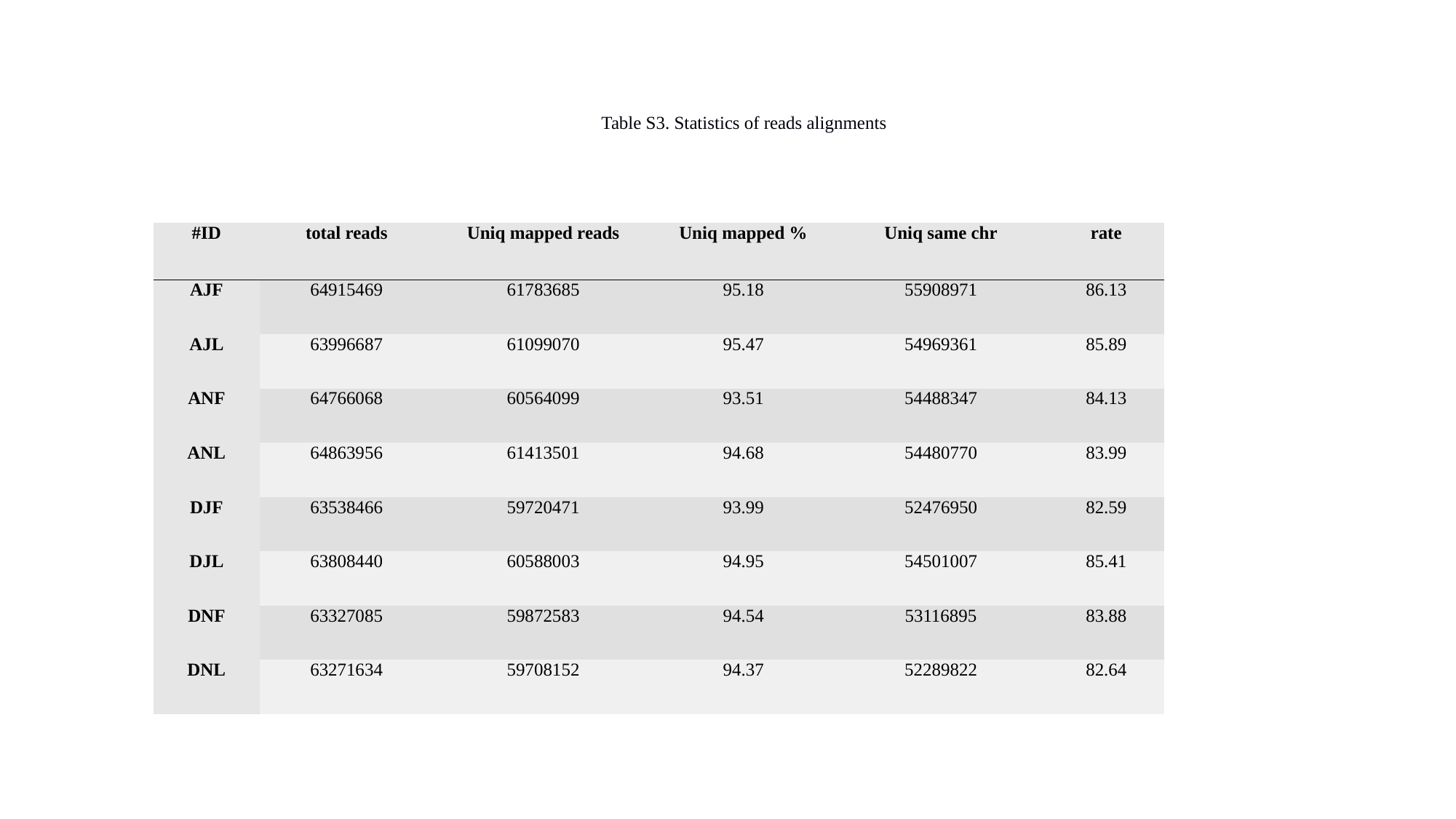

# Table S3. Statistics of reads alignments
| #ID | total reads | Uniq mapped reads | Uniq mapped % | Uniq same chr | rate |
| --- | --- | --- | --- | --- | --- |
| AJF | 64915469 | 61783685 | 95.18 | 55908971 | 86.13 |
| AJL | 63996687 | 61099070 | 95.47 | 54969361 | 85.89 |
| ANF | 64766068 | 60564099 | 93.51 | 54488347 | 84.13 |
| ANL | 64863956 | 61413501 | 94.68 | 54480770 | 83.99 |
| DJF | 63538466 | 59720471 | 93.99 | 52476950 | 82.59 |
| DJL | 63808440 | 60588003 | 94.95 | 54501007 | 85.41 |
| DNF | 63327085 | 59872583 | 94.54 | 53116895 | 83.88 |
| DNL | 63271634 | 59708152 | 94.37 | 52289822 | 82.64 |

## Slide 12
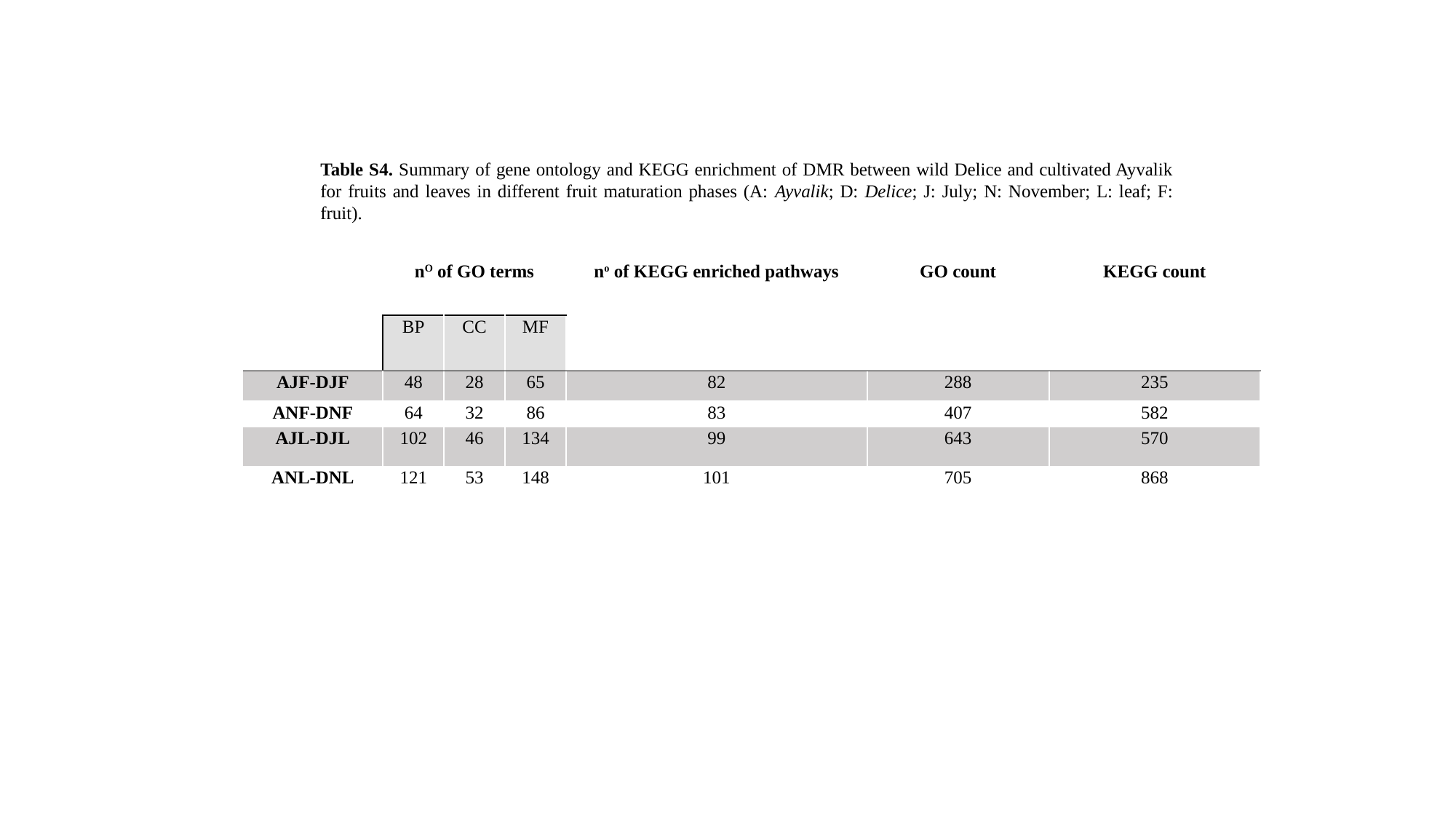

Table S4. Summary of gene ontology and KEGG enrichment of DMR between wild Delice and cultivated Ayvalik for fruits and leaves in different fruit maturation phases (A: Ayvalik; D: Delice; J: July; N: November; L: leaf; F: fruit).
| | nO of GO terms | | | no of KEGG enriched pathways | GO count | KEGG count |
| --- | --- | --- | --- | --- | --- | --- |
| | BP | CC | MF | | | |
| AJF-DJF | 48 | 28 | 65 | 82 | 288 | 235 |
| ANF-DNF | 64 | 32 | 86 | 83 | 407 | 582 |
| AJL-DJL | 102 | 46 | 134 | 99 | 643 | 570 |
| ANL-DNL | 121 | 53 | 148 | 101 | 705 | 868 |

## Slide 13
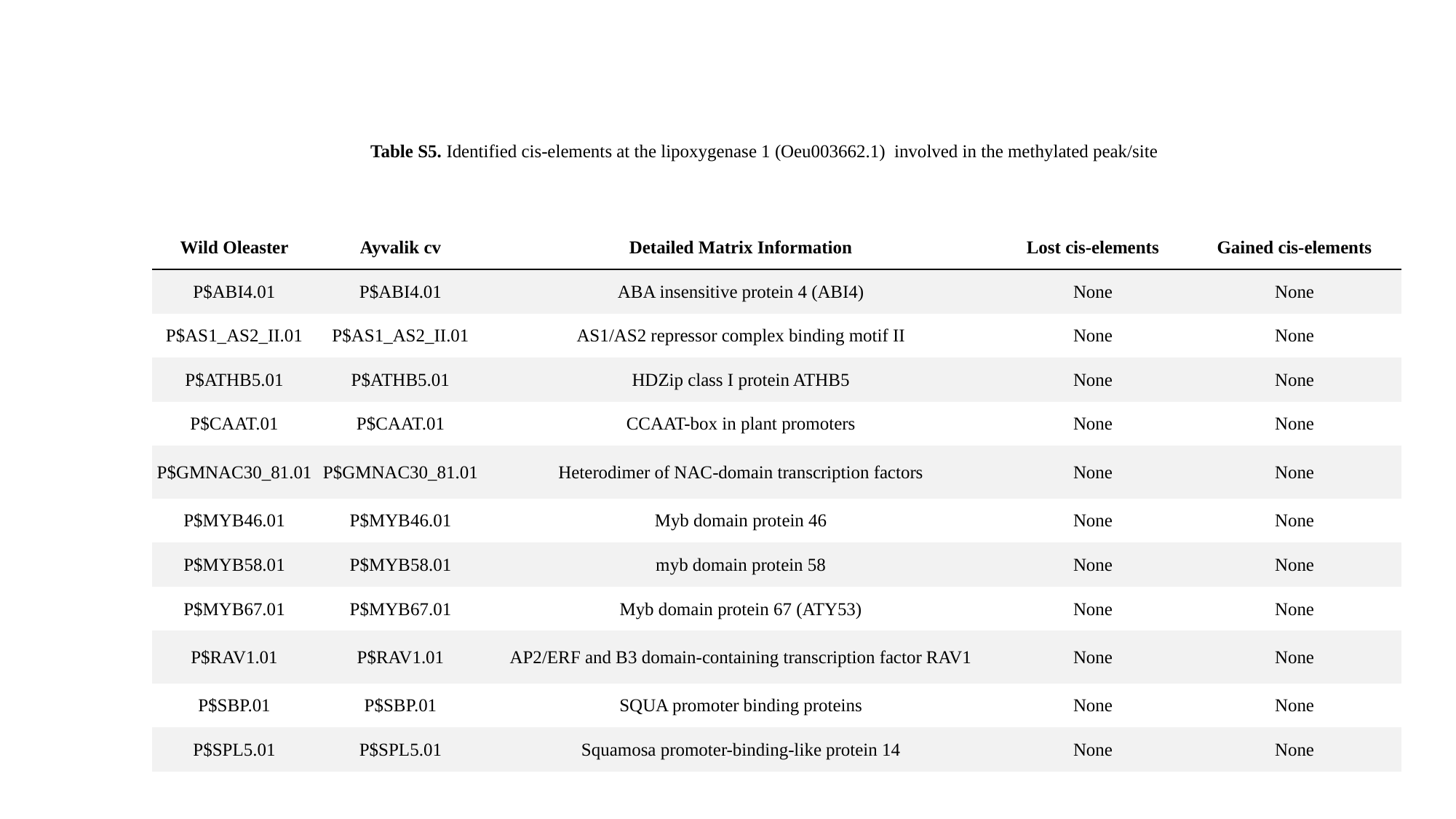

Table S5. Identified cis-elements at the lipoxygenase 1 (Oeu003662.1) involved in the methylated peak/site
| Wild Oleaster | Ayvalik cv | Detailed Matrix Information | Lost cis-elements | Gained cis-elements |
| --- | --- | --- | --- | --- |
| P$ABI4.01 | P$ABI4.01 | ABA insensitive protein 4 (ABI4) | None | None |
| P$AS1\_AS2\_II.01 | P$AS1\_AS2\_II.01 | AS1/AS2 repressor complex binding motif II | None | None |
| P$ATHB5.01 | P$ATHB5.01 | HDZip class I protein ATHB5 | None | None |
| P$CAAT.01 | P$CAAT.01 | CCAAT-box in plant promoters | None | None |
| P$GMNAC30\_81.01 | P$GMNAC30\_81.01 | Heterodimer of NAC-domain transcription factors | None | None |
| P$MYB46.01 | P$MYB46.01 | Myb domain protein 46 | None | None |
| P$MYB58.01 | P$MYB58.01 | myb domain protein 58 | None | None |
| P$MYB67.01 | P$MYB67.01 | Myb domain protein 67 (ATY53) | None | None |
| P$RAV1.01 | P$RAV1.01 | AP2/ERF and B3 domain-containing transcription factor RAV1 | None | None |
| P$SBP.01 | P$SBP.01 | SQUA promoter binding proteins | None | None |
| P$SPL5.01 | P$SPL5.01 | Squamosa promoter-binding-like protein 14 | None | None |

## Slide 14
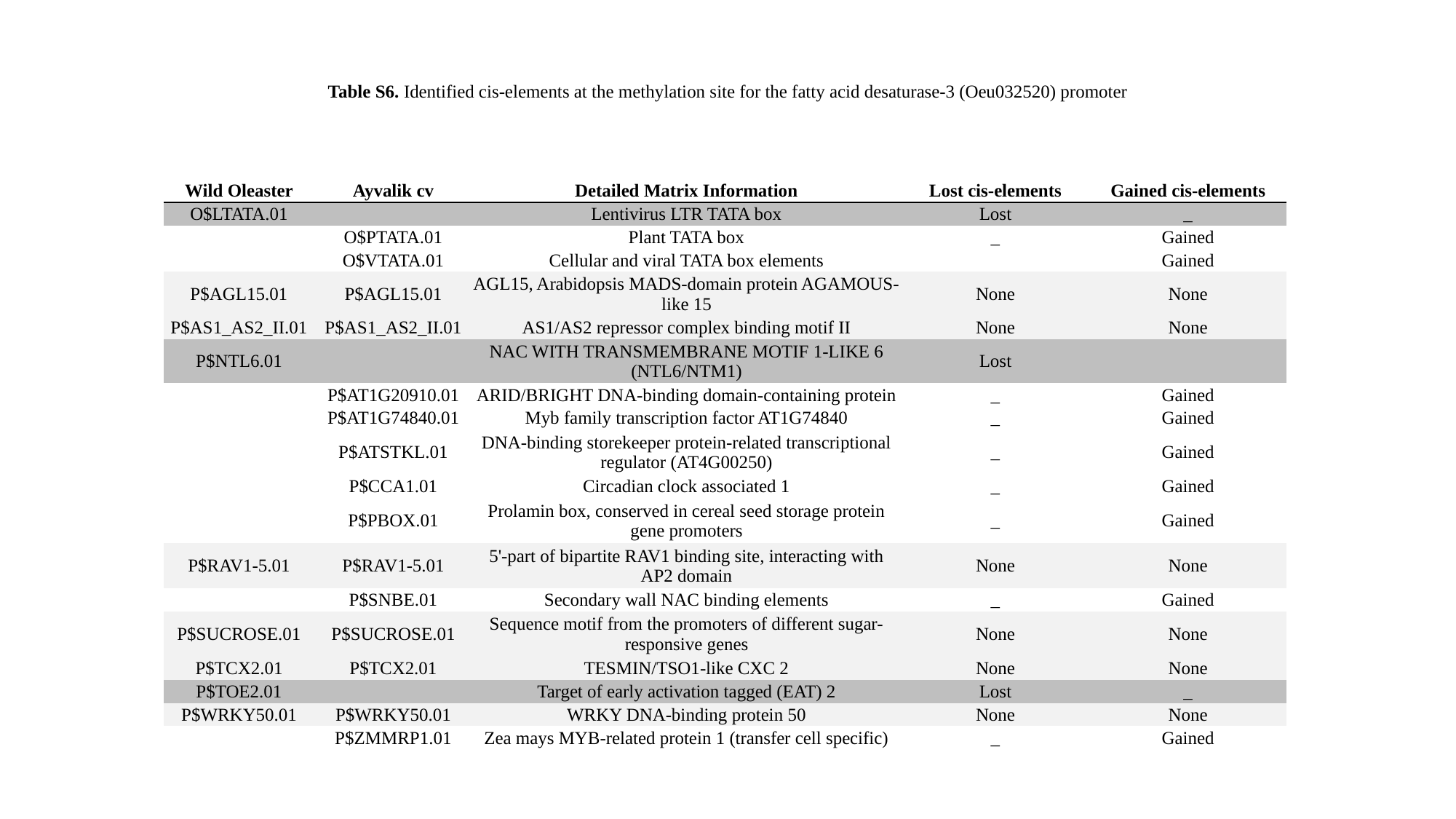

Table S6. Identified cis-elements at the methylation site for the fatty acid desaturase-3 (Oeu032520) promoter
| Wild Oleaster | Ayvalik cv | Detailed Matrix Information | Lost cis-elements | Gained cis-elements |
| --- | --- | --- | --- | --- |
| O$LTATA.01 | | Lentivirus LTR TATA box | Lost | \_ |
| | O$PTATA.01 | Plant TATA box | \_ | Gained |
| | O$VTATA.01 | Cellular and viral TATA box elements | | Gained |
| P$AGL15.01 | P$AGL15.01 | AGL15, Arabidopsis MADS-domain protein AGAMOUS-like 15 | None | None |
| P$AS1\_AS2\_II.01 | P$AS1\_AS2\_II.01 | AS1/AS2 repressor complex binding motif II | None | None |
| P$NTL6.01 | | NAC WITH TRANSMEMBRANE MOTIF 1-LIKE 6 (NTL6/NTM1) | Lost | |
| | P$AT1G20910.01 | ARID/BRIGHT DNA-binding domain-containing protein | \_ | Gained |
| | P$AT1G74840.01 | Myb family transcription factor AT1G74840 | \_ | Gained |
| | P$ATSTKL.01 | DNA-binding storekeeper protein-related transcriptional regulator (AT4G00250) | \_ | Gained |
| | P$CCA1.01 | Circadian clock associated 1 | \_ | Gained |
| | P$PBOX.01 | Prolamin box, conserved in cereal seed storage protein gene promoters | \_ | Gained |
| P$RAV1-5.01 | P$RAV1-5.01 | 5'-part of bipartite RAV1 binding site, interacting with AP2 domain | None | None |
| | P$SNBE.01 | Secondary wall NAC binding elements | \_ | Gained |
| P$SUCROSE.01 | P$SUCROSE.01 | Sequence motif from the promoters of different sugar-responsive genes | None | None |
| P$TCX2.01 | P$TCX2.01 | TESMIN/TSO1-like CXC 2 | None | None |
| P$TOE2.01 | | Target of early activation tagged (EAT) 2 | Lost | \_ |
| P$WRKY50.01 | P$WRKY50.01 | WRKY DNA-binding protein 50 | None | None |
| | P$ZMMRP1.01 | Zea mays MYB-related protein 1 (transfer cell specific) | \_ | Gained |
